# Nrf2–Keap1 Pathway and NLRP3 Inflammasome in Parkinson’s Disease: Mechanistic Crosstalk and Therapeutic Implications

**DOI:** 10.1007/s12035-025-05389-0

**Published:** 2025-11-20

**Authors:** Ridhi Jain, Lalitkumar Vora, Deepak Nathiya, Dharmendra Kumar Khatri

**Affiliations:** 1 Molecular and Cellular Neuroscience Lab, Department of Pharmacology, NIMS Institute of Pharmacy, NIMS University Rajasthan, Jaipur, 303121 India; 2 Department of Pharmacy Practice, NIMS Institute of Pharmacy, NIMS University Rajasthan, Jaipur, 303121 India; 3 School of Pharmacy, Medical Biology Centre, Queen’s University Belfast, 97 Lisburn Road, Belfast, Northern Ireland BT9 7BL UK

**Keywords:** Neuroinflammation, Oxidative stress, NLRP3 inflammasome, Nrf2-Keap1, Parkinson’s disease

## Abstract

Neurodegenerative disorders such as Parkinson’s disease (PD) are characterized by the progressive degeneration of dopaminergic neurons, which is driven primarily by oxidative stress and chronic neuroinflammation. Central to the cellular antioxidant defense system is the nuclear factor erythroid 2–related factor 2 (Nrf2)–Kelch-like ECH-associated protein 1 (Keap1) pathway, which mitigates oxidative damage and preserves mitochondrial integrity. Concurrently, the NOD-like receptor pyrin domain-containing protein 3 (NLRP3) inflammasome acts as a key mediator of innate immune responses and has been increasingly implicated in neuroinflammatory cascades leading to neuronal loss in PD. Emerging evidence indicates a mechanistic interplay between the Nrf2–Keap1 axis and the NLRP3 inflammasome, wherein Nrf2 activation not only counteracts oxidative stress but also suppresses NLRP3-mediated inflammatory signaling. A comprehensive overview of the molecular crosstalk between the Nrf2 and NLRP3 pathways in the pathogenesis of PD, with emphasis on how impaired Nrf2 signaling exacerbates NLRP3 inflammasome activation, is provided. The preclinical and clinical findings on pharmacological agents that activate Nrf2 or inhibit NLRP3 as potential neuroprotective strategies in PD are also discussed. A growing body of evidence underscores the dual therapeutic benefit of targeting oxidative stress and inflammation via Nrf2 inducers and NLRP3 inhibitors. Nonetheless, obstacles such as restricted blood–brain barrier permeability, unintended effects, and variable clinical trial outcomes hinder the application of these findings in clinical settings. The advancement of disease-modifying therapies for PD hinges on continuous research aimed at deepening the mechanistic understanding of the Nrf2–NLRP3 axis and refining pharmacological strategies.

## Introduction

**Fig. 1:**
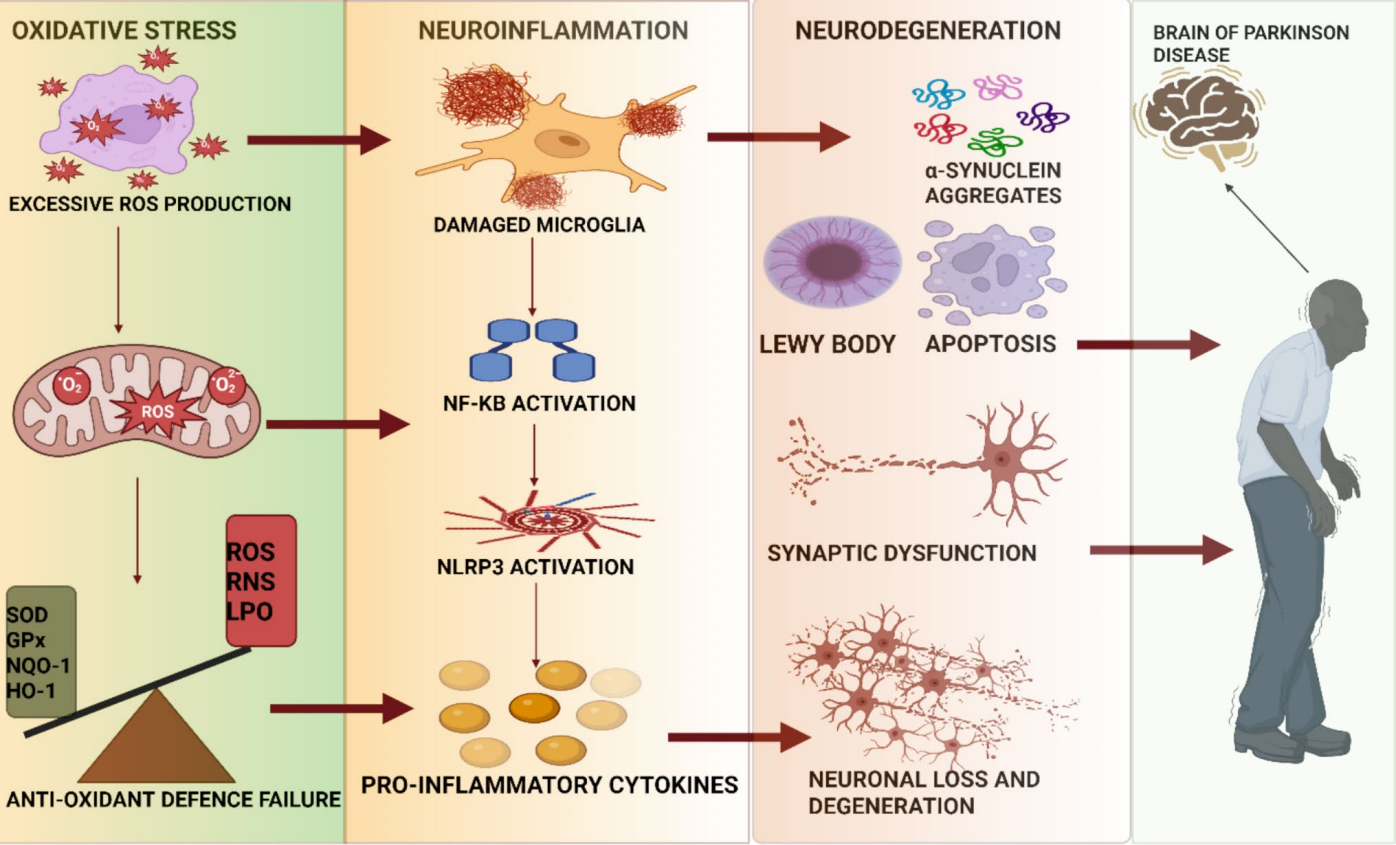
Oxidative stress–neuroinflammation–neurodegeneration axis in Parkinson’s disease. Schematic representation illustrating the interconnected cascade from oxidative stress to neuroinflammation and neurodegeneration, which is organized into three concentric stages. The innermost layer depicts the initiation of oxidative stress, which is driven primarily by excessive reactive oxygen species (ROS) production due to mitochondrial dysfunction and impaired antioxidant defense systems (e.g., SOD, GPx, NQO1, and HO-1). Persistent oxidative stress triggers the activation of microglia, promoting their polarization toward a proinflammatory phenotype. Activated glial cells secrete proinflammatory cytokines, while ROS further potentiate inflammatory signaling through key mediators such as NF-κB and the NLRP3 inflammasome. This establishes a feed-forward loop of chronic neuroinflammation and oxidative damage. The outermost stage illustrates the downstream consequences of sustained inflammation, including neuronal apoptosis, α-synuclein aggregation, synaptic impairment, and axonal degeneration. Collectively, the diagram highlights a self-perpetuating cycle of oxidative stress and neuroinflammation that accelerates the loss of dopaminergic neurons, a hallmark of Parkinson’s disease and other neurodegenerative disorders

Neurodegenerative disorders impose an escalating public health burden, with profound personnel, social and economic consequences [1]. PD affects more than 8.5 million people worldwide ranking as the second most common neurodegenerative disorder after Alzheimer’s disease, but the fastest-growing in terms of prevalence, disability and mortality rates. This surge is driven by population aging, increased life expectancy, regional demographic transitions and projections suggest the global PD population may exceed 25 million by 2050 [2]. A movement disorder with classic parkinsonian motor symptoms is caused by a shortage of dopamine in the basal ganglia. Clinically, these motor impairments such as bradykinesia, rigidity and tremors arise from the progressive loss of dopaminergic neurons in the substantia nigra pars compacta (SNpc) a pathological hallmark of PD [3]. This neuronal loss arises from a complex interplay of genetic predisposition and environmental exposures, which ultimately converge on a common set of molecular disturbances. Current evidence supports a multifactorial etiology in which more than 90 susceptibility loci, rare monogenic variants (e.g., LRRK2, SNCA, PRKN, and PINK1) and environmental toxicants (pesticides, heavy metals, and solvents) converge on three inter-related pathological hallmarks: oxidative stress, mitochondrial dysfunction and chronic neuroinflammation. Among these mechanisms, oxidative stress emerges as a central driver of neuronal vulnerability not only by directing damaging proteins, lipids and nucleic acids but also by perpetuating inflammatory signaling within the nigrostriatal pathway. Elevated iron in the SNpc, reduced glutathione, and widespread oxidation of lipids document the oxidative milieu in post-mortem PD brains, whereas deficits in complex I activity and impaired mitophagy underscore the pivotal role of dysfunctional mitochondria in reactive oxygen species (ROS) overload. Accordingly, cellular oxidative stress is facilitated by the iron-catalyzed Fenton reaction and the degradation of lipid-derived alkoxy and peroxy radicals, and PD brains have a relatively high concentration of iron in the substantia nigra. Since mitochondria are thought to be the main source of reactive oxygen species (ROS), dysfunctions in these organelles lead to redox alterations in disease [4]. Persistent oxidative imbalance amplifies microglial activation, which in turn, engages innate immune sensors such as NOD-like receptor pyrin domain-containing 3 (NLRP3) a multiprotein complex that links mitochondrial damage and α-synuclein aggregation to the maturation of interleukin-1β and interleukin-18. Hyper-activation of NLRP3 is consistently observed in PD patient samples and experimental models, and its genetic or pharmacological inhibition mitigates dopaminergic neurodegeneration. Persistent activation of microglia can sustain a cycle of inflammation and oxidative stress, amplify neuronal vulnerability and accelerate neurodegeneration in PD [5]. Breaking this self-perpetuating cycle requires the activation of the endogenous defense system that counteracts oxidative and inflammatory stressors. Opposing this inflammatory axis is the Nrf2–Keap1 pathway, the master transcriptional regulator of cellular antioxidant and detoxification programs [6]. Nrf2 is a protein containing 605 amino acids that regulates the expression of antioxidants and genes. Under basal conditions, Nrf2 is sequestered by Keap1 and undergoes proteasomal degradation [7]. However, electrophilic stress disrupts this interaction, allowing Nrf2 to translocate into the nucleus, energizing the transcriptional activity of antioxidant enzymes such as heme oxygenase-1 (HO-1), NADPH quinone oxidoreductase-1 (NQO1), and glutathione peroxidase (GPx) [8]. Nrf2 deficiency exacerbates nigrostriatal neurodegeneration, increases lipid peroxidation and augments glial activation in PD models, highlighting its neuroprotective capacity. In contrast, the NLRP3 inflammasome is a key mediator of the neuroinflammation process [9]. As a multiprotein complex activated by diverse stressors, including mitochondrial damage, NLRP3 triggers the release of pro-inflammatory cytokines [10]. Persistent activation of NLRP3 contributes to the chronic neuroinflammatory state and exacerbates neuronal damage [11]. The NLRP3 inflammasome plays a key role in transmitting inflammatory signals and damaged components to mitochondria, intensifying neuroinflammation in PD [12]. However, Nrf2 regulates the inflammasome and protects mitochondria by reducing oxidative stress and promoting mitochondrial health [13]. Mounting data reveal intimate Nrf2–NLRP3 crosstalk: Nrf2 activation limits the transcriptional priming of NLRP3, curtails mitochondrial ROS, and directly interferes with inflammasome assembly, whereas unchecked NLRP3 signaling feeds forward to inhibit Nrf2 and perpetuate oxidative damage (sciencedirect.com). This reciprocal regulation creates a pathogenic loop whose dysregulation accelerates neuronal death [14]. Activation of the Nrf2-Keap1 pathway can inhibit NLRP3 and act as a protective mechanism in Parkinson’s disease [15]. A three-stage model of dopaminergic neurodegeneration, including oxidative stress, inflammation, and cell death, is depicted in Fig. 1. This review synthesizes recent molecular insights into how Nrf2–Keap1 signaling governs NLRP3 inflammasome activity, delineates the consequences of axis dysregulation for mitochondrial integrity and dopaminergic survival and critically appraises emerging pharmacological approaches aimed at restoring this balance to modify PD progression.

## The Nrf2-Keap1 Pathway: Structure, Mechanisms, and Regulation

The molecular architecture of Nrf2-Keap1 reflects its critical role in oxidative stress regulation. Nrf2 is a multidomain transcription factor composed of seven highly conserved domains (Neh1–Neh7) that regulate its stability, interaction with Keap1, and transcriptional activity [16]. The Neh2 domain, which contains ETGE and DLG motifs, is pivotal for Keap1 binding and ubiquitination, whereas the Neh1 domain features a basic leucine zipper (bZIP) structure essential for DNA binding and dimerization with small Maf proteins [17]. Additional domains, such as Neh4 and Neh5, recruit the coactivators CREB-binding protein (CBP) and repressor-associated coactivator (RAC) for transcription, whereas the Neh6 and Neh7 domains modulate Nrf2 degradation and repression respectively [18]. Keap1, a homodimeric protein, acts as a substrate adaptor for the Cullin-3 ubiquitin E3 ligase complex. Its structure includes the Broad Complex Tram track and Bric-a-Brac (BTB) domain for dimerization and Cul3 interaction, the intervening region (IVR) containing redox-sensitive cysteine residues (e.g., Cys151) and the Kelch domain, which binds the ETGE and DLG motifs of Nrf2 [19]. Nrf2 is the master regulator of redox homeostasis under basal conditions and is tightly regulated by Keap1. Keap1 binds to the Neh2 domain of Nrf2 via its high-affinity ETGE and low-affinity DLG motifs via a “hinge and latch” mechanism [20]. This position of Nrf2 for polyubiquitination by the Cul3-RBX-1 complex marks it for proteasomal degradation, maintaining a low level of Nrf2 under normal conditions. Keap1 contains highly reactive cysteine residues, including Cys151, Cys273, and Cys288, which are critical for its functional sensitivity to stress [21]. These residues are located in distinct structural domains of Keap1, including the BTB, IVR and Kelch domains. Upon exposure to electrophilic stress, these cysteine residues undergo various modifications, such as S-alkylations, S-nitrosylation, S-glutathionylation, and S-sulfhydration [22]. For example, electrophiles such as sulforaphane and tert-butyl hydroquinone (tBHQ) can covalently modify these cysteine residues, leading to conformational changes in Keap1 [23]. The most studied cysteine modification is Cys151 in the BTB domain, which disrupts the interaction of Keap1 with the Cul3 ligase complex. This modification prevents Nrf2 from being ubiquitinated and degraded [24]. Similarly, modifications at Cys273 and Cys288 in the IVR region impair the ability of Keap1 to act as an adaptor substrate for ubiquitination. As a result, Nrf2 escapes proteasomal degradation and translocates to the nucleus. Inside the nucleus, it binds to the ARE in the promoter region of its target genes, driving the expression of cryoprotective proteins such as HO-1, NQO1, and glutathione S-transferase [25]. Nrf2 is tightly regulated in numerous ways, among which phosphorylation by kinases plays a crucial role in the posttranslational regulation of Nrf2. Protein kinase C (PKC) is phosphorylated at Ser40 within the Neh2 domain, disrupting the Keap1 interaction of Nrf2 [26]. Casein kinase 2 (CK2) and AMP-activated protein kinase 2 (AMPK2) positively regulate Nrf2 [27]. To facilitate its interaction with the β-transducin repeat-containing protein (β-Trcp), the Cul1 E3 ubiquitin ligase complex, which negatively controls Nrf2, glycogen synthase kinase-3 (GSK-3) phosphorylates a particular serine residue within the Neh6 domain [28]. The other mechanism of Nrf2 regulation is mediated by p62 or a sequence. p62, a selective autophagic receptor, plays a critical role in this process through the Keap1 sequestration mechanism. In addition to oxidative stress-mediated activation, p62 can also activate Nrf2 in a redox-independent manner. In this pathway, p62 binds to Keap1 via its Keap1-interacting region (KIR) and recruits it to the autophagosome through the LC3-interacting region (LIR), leading to Keap1 degradation in autolysosomes [29]. This degradation decreases the Keap1 level in the cytoplasm, relieving its inhibition of Nrf2 and promoting its nuclear translocation and stabilization. This effect has been demonstrated in models employing p62 overexpression, independent of oxidative stress [30]. Inside the nucleus Nrf2 activates the expression of antioxidant genes, including HO-1, NQO1, superoxide dismutase (SOD), and GPx. HO-1 degrades pro-oxidant heme into biliverdin, carbon monoxide and free iron (Fe2 +), reducing oxidative stress by removing heme. Biliverdin is converted into bilirubin, a strong antioxidant, whereas carbon monoxide has anti-inflammatory properties [31]. NQO-1 prevents redox cycling of quinones, thereby reducing ROS production. However, during this process, free iron is liberated and quickly sequestered by ferritin to stop iron-mediated ROS generation via the Fenton reaction [32]. SOD and GPx further neutralize superoxide radicals and hydrogen peroxide. By activating these enzymes, Nrf2 plays a pivotal role in maintaining mitochondrial integrity, improving energy production and preventing the oxidative damage associated with neurodegenerative diseases such as Parkinson’s disease.

## NLRP3 Inflammasome Structure and Its Activation

The NLRP3 inflammasome is essential to the innate immune system, which provides defense against infections caused by bacteria, fungi, and viruses. The NLRP3 inflammasome also recognizes damage-associated molecular patterns (DAMPs), including ATP, uric acid crystals, silica, asbestos, alum, and protein deposits [12]. NLRP3 consists of the adaptor protein apoptosis-associated speck-like protein (ASC) with a caspase recruitment domain (CARD) 8 and the effector pro-caspase-1 protein, which makes up the NLRP3 multiprotein complex. It contains three primary domains: a pyrin domain (PYD) for ASC interaction, a NACHT domain for oligomerization and leucine-rich repeats (LRRs) for sensing activation signals [33]. ASC is an adaptor protein that contains a PYD to bind to NLRP3 and a CARD to interact with procaspase-1. Caspase-1, which is synthesized as an inactive zymogen, is recruited to the inflammasome via ASC. Upon activation, NLRP3 oligomerizes and recruits ASC via PYD-PYD binding, forming a scaffold that transports procaspase-1 molecules into proximity through the CARD-CARD interaction. This proximity induces caspase-1 autocleavage and activation, enabling downstream processing of proinflammatory cytokines [34]. In PD, mitochondrial ROS, extracellular ATP and α-synuclein aggregates act as key triggers for NLRP3 activation. Mitochondrial ROS, a result of mitochondrial dysfunction, signals cellular stress and releases mitochondrial DNA (mtDNA), amplifying activation. Extracellular ATP, often released from damaged cells, binds to the P2X7 receptor, inducing potassium efflux and leading to NLRP3 activation [35]. Additionally, α-synuclein disrupts lysosomes and mitochondria, promoting inflammasome activation. Once activated, NLRP3 facilitates caspase-1 activation, which results in the conversion of pro-IL-1β and pro-IL-18 into their active forms, IL-1β and IL-18 [36]. These cytokines drive chronic neuroinflammation, exacerbating microglial activation and neuronal loss and creating a vicious cycle of inflammation and neurodegeneration in PD.

### Various Regulatory Mechanisms of NLRP3

NLRP3 is tightly regulated at multiple levels, including the DNA level; transcriptional, posttranslational, and metabolic mechanisms; and noncoding RNA and ion flux modulation, ensuring the precise control of its activation [37]. Transcriptionally, NF-κB is a primary driver that induces NLRP3 expression through Toll-like receptor (TLR) signaling in response to ligands such as lipopolysaccharides (LPS), while STAT proteins further modulate transcription. Posttranslational modifications (PTMs) critically fine-tune NLRP3 activity [38]. K48-linked ubiquitination promotes proteasomal degradation, whereas K63-linked ubiquitination activates NLRP3 by recruiting binding partners, and deubiquitination by enzymes such as BRCA1/BRCA2-containing complex subunit 3 (BRCC3) and ubiquitin-specific protease 1 USP1/UAF1 or BRCC3 reverses this inhibition. Phosphorylation also regulates NLRP3, with casein kinase 2 (CK2) inhibiting its activation, whereas JNK- and PKC-mediated phosphorylation promotes it [39]. Sumoylation represses NLRP3 activation and can be reversed by the Sentrin/SUMO-specific protease (SENP) protease [40]. Protein–protein interactions are essential, with TXNIP facilitating NLRP3 activation under oxidative stress, with NEK7 being critical for inflammasome assembly via interaction with the LRR domain and with HSP90/SGT1 stabilizing the inactive form [41]. Metabolic cues also influence NLRP3; glycolysis enhances its activation by encouraging mitochondrial dysfunction, whereas AMPK activation suppresses it by inducing autophagy and preserving mitochondrial integrity [42]. The mTOR pathway activates NLRP3 by driving anabolic processes. ROS, especially mitochondrial ROS (mtROS), serve as potent activators by releasing danger-associated molecular patterns (DAMPs), while the antioxidant N-acetylcysteine (NAC) mitigates this activation [43]. Ion flux regulation is another factor involved in potassium efflux, elevated intracellular calcium, and chloride efflux, all of which serve as activation signals [44]. By binding to their target, the NLRP3 gene, a variety of noncoding RNA products, including microRNAs (miRNAs) and long noncoding RNAs (lncRNAs), can control the expression of the NLRP3 inflammasome at the posttranscriptional level. A number of miRNAs, including miR-7 [45], miR-22 [46], miR-30e [47], miR-133b [48], and miR-223 [49], block the NLRP3 inflammasome. Pharmacological inhibitors such as MCC950, which inhibit NLRP3 ATPase activity [50] and CY-09 which prevents its activation, and Bay-11 7082 which targets NF-κB and offers therapeutic options [51]. Finally, epigenetic regulation via histone modification, such as acetylation of methylation at the NLRP3 promoter and DNA methylation, silences NLRP3 expression and further refines its transcriptional control, highlighting the intricate regulation of this critical inflammasome component [52].

## Nrf2-Keap1 Pathway and NLRP3 in PD: A Mechanistic Crosstalk

This section breaks down the molecular relationship between Nrf2-Keap1 signaling and NLRP3 inflammasome activity in Parkinson’s disease into multiple subsections to provide a more comprehensive and understandable picture. By focusing on a different aspect of the crosstalk—from its importance in PD pathogenesis and cell-type-specific contributions to well-defined molecular suppression mechanisms and auxiliary regulatory pathways, each promises both thematic clarity and mechanistic depth.

### Overview of Mechanistic Crosstalk in PD Pathogenesis

PD is caused by a number of disease-specific stressors, including aggregation of α-synuclein, dopamine oxidation products, mitochondrial dysfunction, and exposure to environmental neurotoxins such as rotenone and MPTP. These stressors result in the release of mitochondrial danger signals such as cardiolipin and mtDNA, as well as an excess of mitochondrial reactive oxygen species (mtROS). These mitochondrial DAMPs and oxidants prime and activate the microglial NLRP3 inflammasome assembly, drive caspase-1 cleavage, and mature IL-1β/IL-18, all of which contribute to the maintenance of neuroinflammation in the substantia nigra. In addition to these intracellular inflammatory events, the integrity of the blood–brain barrier (BBB) plays a decisive role in shaping the neuroinflammatory environment in PD [53]. An intact BBB reduces oxidative damage to dopaminergic neurons and microglial priming by blocking the entry of pro-inflammatory cytokines into the brain parenchyma. However, BBB rupture, which has been observed in both PD patients and experimental models, encourages the entry of inflammatory mediators and oxidative agents, which raises NLRP3 activation and speeds up neuronal degradation [54]. Therefore, in PD Nrf2 activation and BBB preservation may cooperate to fight oxidative and inflammatory stressors [55]. By triggering NLRP3 activation in human induced pluripotent stem cells (iPSC)-derived microglia through TLR2 engagement and mitochondrial damage, α-synuclein species have been shown to directly contribute to innate immunological activation in PD [56, 57]. The Nrf2-keap1 system halts this inflammatory axis by finding redox imbalance through electrophilic or oxidative changes to Keap1 cysteines. It not only stabilizes Nrf2, but it also activates a number of cytoprotective genes (HO-1, NQO1, GCLC/GCLM, and other phase-II enzymes) that protect cells from lipid peroxides, restore glutathione balance, and keep mitochondria healthy. By lowering intracellular ROS and inhibiting NF-κB-dependent transcriptional priming of NLRP3, Nrf2 activity functionally lowers inflammasome formation in sterile neuroinflammatory conditions such as PD. However, when mitochondrial quality control (MQC) mechanisms, particularly PINK1/Parkin-dependent mitophagy, are compromised, damaged mitochondria accumulate, mtROS and mtDNA leak into the cytosol, and Nrf2-driven defenses are overloaded [18]. This shifts the balance in favor of NLRP3 activation. Ferroptosis (iron-dependent lipid peroxidation) is also implicated as a mechanistic bridge by emerging evidence: GPX4 dysfunction and iron accumulation increase lipid peroxides, which sensitize neurons to ferroptotic death and provide extra DAMP-like signals that increase inflammasome activation [58, 59]. When combined, ferroptotic lipid peroxidation and MQC failure seem to be proximal upstream factors that establish the Nrf2-NLRP3 rheostat in PD. As such, they are appealing targets for therapies aimed at reestablishing redox-inflammatory homeostasis.

### Microglial Activation in Nrf2-NLRP3 Signaling During PD

The inflammatory aspect of Parkinson’s disease pathogenesis is mostly mediated by microglia, the CNS’s resident immune cells. Aggregated α-synuclein fibrils, dopamine-derived quinones, and mitochondrial DAMPs, including cardiolipin and mtDNA, are strong inducers of microglial NLRP3 inflammasome activation in PD. By encouraging the development of IL-1β and IL-18 in a caspase-1-dependent manner, this activation intensifies neuroinflammatory cascades and fosters an environment that is antagonistic to dopaminergic neurons [60, 61]. Microglia can change from pro-inflammatory (M1-like) to anti-inflammatory or reparative (M2-like) phenotypes, demonstrating their extraordinary adaptability. An important factor in this polarization is Nrf2 activity: strong Nrf2 signaling inhibits NF-κB-mediated NLRP3 priming, boosts antioxidant defenses via HO-1, NQO1, and glutathione formation, and encourages a change to an M2-like phenotype that aids in tissue regeneration [62]. On the other hand, aging, genetic predisposition, and persistent oxidative stress can all hinder Nrf2 activation in microglia, allowing for prolonged NLRP3 activity and sustaining the inflammatory cycle [63]. Microglial behavior is further influenced by interactions with astrocytes; astrocytic Nrf2 activation can reduce microglial inflammasome activity and buffer extracellular oxidative stress, underscoring the significance of glial-glial communication in controlling neuroinflammation in PD [64].

### Mechanism Involving Nrf2-Keap1 Complex Mitigates the NLRP3 Inflammasome in PD

**Fig. 2:**
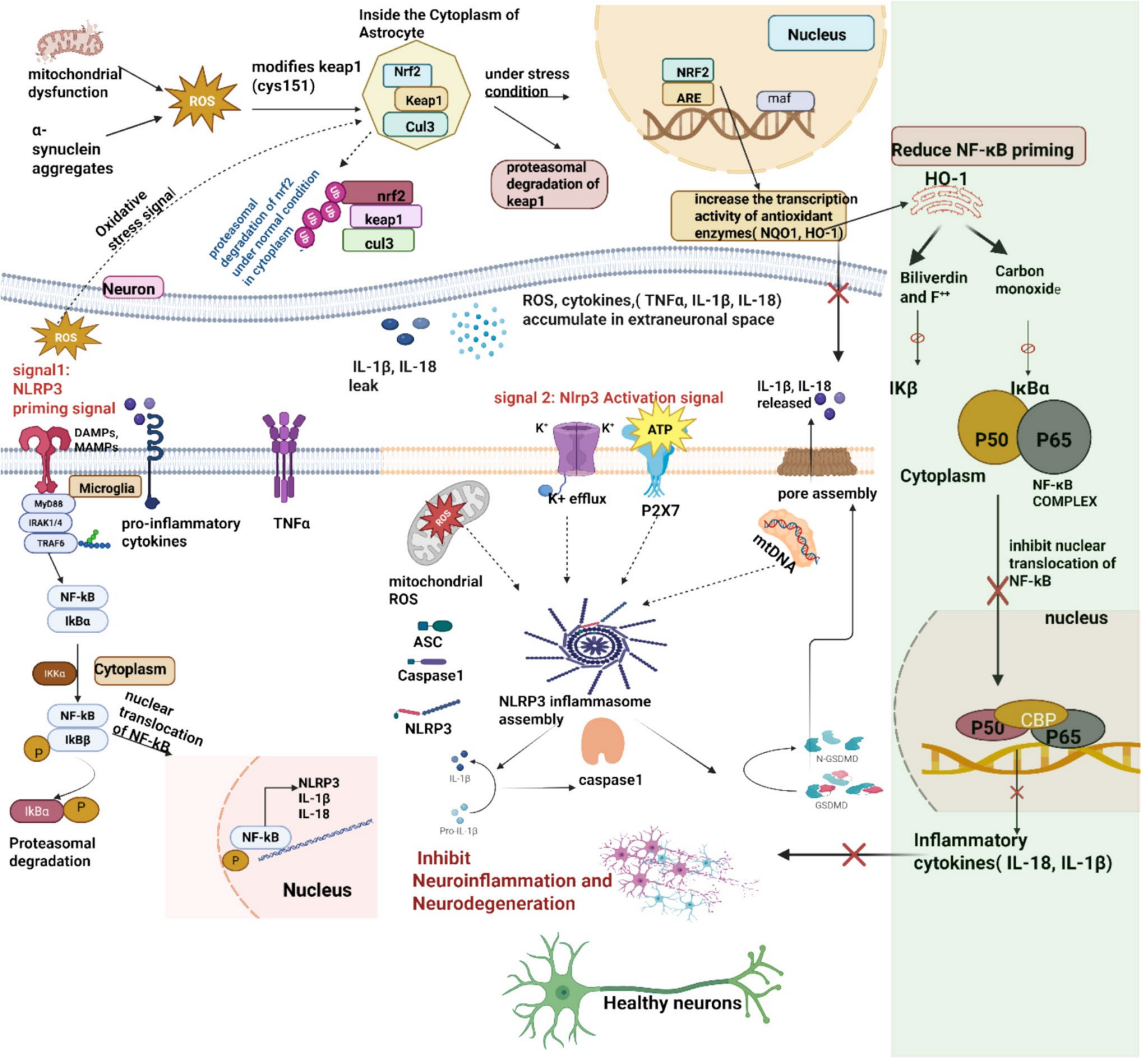
Crosstalk between the Nrf2–Keap1 axis and NLRP3 inflammasome activation. The diagram illustrates the two canonical steps of NLRP3 inflammasome signaling in microglia and how they are modulated by the Nrf2–Keap1 antioxidant pathway primarily in astrocytes. [1] Priming: danger‑ or microbe‑associated molecular patterns (DAMPs/MAMPs) engage pattern‑recognition receptors, activating the IKK complex. Phosphorylation‑dependent degradation of IκB liberates the NF‑κB (p50/p65) dimer, which translocates to the nucleus and up‑regulates the expression of the NLRP3, pro‑IL‑1β and pro‑IL‑18 transcripts. [2] Activation: secondary stimuli—including extracellular ATP, mitochondrial ROS and K⁺ efflux—promote NLRP3 oligomerization, the recruitment of ASC and pro‑caspase‑1, and the autoproteolytic processing of caspase‑1. Active caspase‑1 cleaves pro‑IL‑1β and pro‑IL‑18 into their mature, secreted cytokines, driving a robust inflammatory response. In astrocytes, oxidative stress disengages Nrf2 from its cytoplasmic repressor Keap1, allowing Nrf2 to accumulate in the nucleus, bind antioxidant‑response elements (AREs) and induce the expression of cytoprotective genes such as HO‑1 and NQO1. Heme oxygenase‑1 degrades heme to biliverdin, carbon monoxide (CO) and free iron. Biliverdin and CO exert anti‑inflammatory effects by scavenging ROS and interrupting NF‑κB signaling: CO stabilizes IκBα/β, thereby retaining NF‑κB in the cytosol and diminishing the transcription of inflammasome‑related genes. This Nrf2‑driven feedback loop attenuates NLRP3 assembly, limits caspase‑1 activation and ultimately restrains IL‑1β/IL‑18 maturation, preserving cellular homeostasis

In PD, the NLRP3 inflammasome acts as a central driver of neuroinflammation, perpetuating a self-sustaining inflammatory loop that accelerates the degeneration of dopaminergic neurons. Excess ROS accumulates through multiple PD-related mechanisms, including mitochondrial complex 1 impairment, spontaneous dopamine oxidation and chronic exposure to neurotoxic environmental agents. These ROS are not merely damaging by-products but also serve as signaling intermediates that influence both protective and pathological pathways. A key protective mechanism involves the oxidation of redox-sensitive cysteine residues on Keap1 notably Cys151, Cys273, and Cys288. This structural modification disrupts the Keap1-cullin-3 ubiquitin ligase complex liberating Nrf2 and allowing its stabilization and migration into the nucleus [65]. Once nuclear, Nrf2 dimerizes with small Maf proteins and binds to ARE to initiate the transcription of genes that strengthen the cells’ antioxidant capacity. Two of the most prominent products are HO-1 and NQO-1. HO-1 catalyzes the breakdown of pro-oxidant heme into biliverdin, carbon monoxide (CO) and ferrous ion (Fe2 +). Biliverdin is rapidly converted to bilirubin, a lipophilic antioxidant capable of scavenging lipid peroxyl radicals within neuronal membranes. CO exerts an anti-inflammatory effect by inhibiting IκB (IKK) kinase activity, thereby stabilizing IκBα and preventing NF‑κB from translocating to the nucleus, which reduces the transcriptional priming of NLRP3 and pro-inflammatory cytokines. Concurrently Fe2 + is rapidly sequestered by ferritin, limiting its involvement in the Fenton reaction that generates hydroxyl radicals and contributes to iron-driven oxidative injury in the dopaminergic neurons [66]. NQO1 supports the antioxidant network by catalyzing the two-electron reduction of quinones to hydroquinone, thereby bypassing semiquinone intermediates that can undergo redox cycling. This reaction holds the continuous regeneration of superoxide and hydrogen peroxide, breaking the oxidative stress amplification loop. Together, HO-1 and NQO1 activity lowers the intracellular ROS levels, preserves mitochondrial membrane potential, and reduces the release of DAMPS such as cardiolipin and mitochondrial DNA, both potent activators of NLRP3 [67], as shown in Fig. 2.

Through the combined suppression of priming signals and inhibition of activation cues, Nrf2 limits NLRP3 assembly, caspase 1 activation and maturation of IL-1β beta and IL-18 [68]. This is particularly relevant in PD where sustained microglial activation in inflammasome signaling intensifies oxidative stress, disrupts synaptic integrity and accelerates dopaminergic cell loss. The ROS–Nrf2–NLRP3 network thus highlights the dual nature of ROS in PD: transient, regulated increase can trigger protective antioxidant responses, whereas persistent ROS elevation fuels inflammasome activity and neurodegeneration. By integrating oxidative stress sensing with inflammatory control, Nrf2 emerges as a pivotal regulatory node and therapeutic target in PD [69]. The detailed insight of pre-clinical findings involving the effect of Nrf2–Keap1 on NLRP3 is shown in Table 1.

**Table 1:** Insights from preclinical studies: Effects of Nrf2 activators on NLRP3 in toxin-induced PD models

Nrf2 activators	Neurotoxin-induced	Study type	Outcome	Reference
Baicalein	MPTP (1-Methyl-4-phenyl-1,2,3,6-tetrahydropyridine)	In vivo: C57BL/6 mice	Inhibited caspase1, reduce NLRP3/caspase1 pathway	[70]
Sesamol, thymol	Manganese-induced PD	In vivo: adult male Sprague–Dawley rats	modulating TLR4/NLRP3/NF-κB, GSK-3β, Nrf2/HO-1,	[71]
DMF	MPTP, LPS	In vivo: C57BL/6 miceIn vitro: BV-2 cell line	Nrf2 deficiency increases NLRP3 activation	[72]
Supercurcumin, carnosol, cobalt protoporphyrinix	LPS (Lipolysaccharide)	In vitro: BV-2 cell line	Decrease TNF-α enhanced HO-1 and bilirubin levels	[73]
Astrogaloside IV	MPTP	In vivo: C57BL/6 miceIn vitro: BV-2 cell line	Inhibited NF-κB mediated NLRP3 activation and activated Nrf2	[74]
Tert-butyl hydroquinone and GKT136901	LPS	In vitro: mixed glial cultureIn vivo: C57BL/6 mice, NOX4 KO mice, C57BL/6J mice, Nrf2 KO mice	TBHQ reduced nlrp3 expression and GKT36901 decreased IL-1β release.	[75]
Piperine	MPTP	In vivo: Male C57BL/6 mice,	Number of activated microglia, expression of cytokine IL-1β decreased	[76]
Dimethyl itaconate	LPS	In vivo: C57BL/6 miceIn vitro: BV-2 cell line	Decreased NLRP3 assembly, LDS release, NF-κB phosphorylation and ROS	[77]
Carvacrol	Rotenone	In vivo: Swiss albino mice	Alleviated oxidative stress and decreased cytokines	[78]
Celastrol	MPTP	In vivo: C57BL/6 mice	Inhibited NLRP3 inflammasome	[79]
*Nardostachys jatamansi* and levodopa	Rotenone	In vivo: Sprague–Dawley rats	inhibiting NLRP3, activating Nrf2	[80]
*Uncaria rhynchophylla*	MPTP	In vivo: C57BL/6 mice	Inhibited NLRP3/caspase1 pathway	[81]

### *Role of Autophagy (*via* p62) Degrading NLRP3 in PD*

**Fig. 3:**
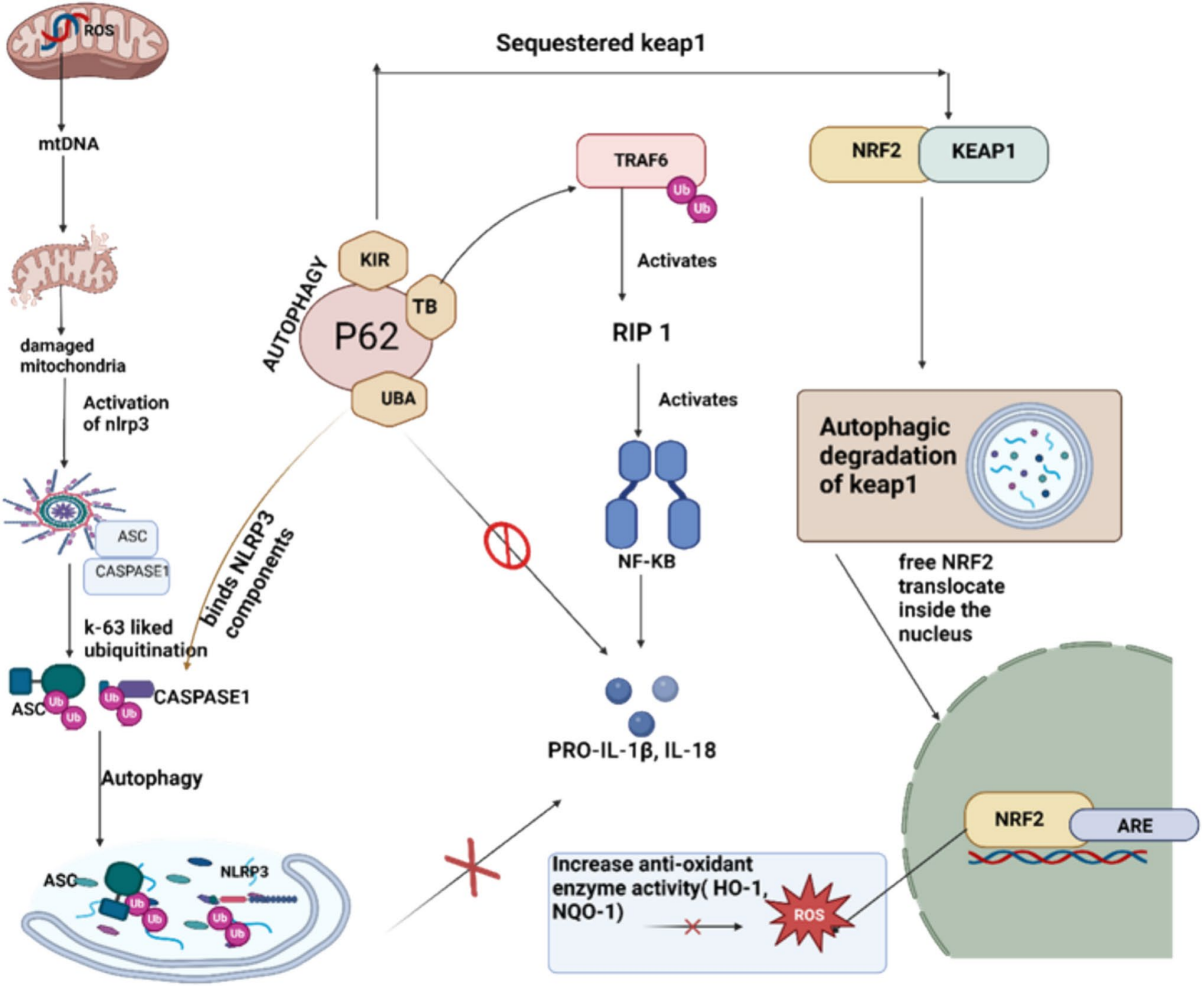
Multifaceted regulatory role of p62 in autophagy, inflammation, and redox homeostasis. This schematic illustrates the integrative role of p62 (also known as sequestosome 1) in modulating the NLRP3 inflammasome, NF-κB signaling, and the Nrf2–Keap1 antioxidant pathway under conditions of cellular stress. Under inflammatory or oxidative stress, NLRP3 inflammasome components—including NLRP3, ASC, and caspase-1—undergo K63-linked polyubiquitination, marking them for selective autophagic degradation. p62 recognizes these polyubiquitinated proteins via its ubiquitin-associated (UBA) domain and facilitates their aggregation and sequestration into autophagosomes. Subsequent autophagic degradation of these components suppresses inflammasome assembly, thereby limiting the maturation and release of IL-1β and IL-18. In parallel, p62 modulates inflammatory signaling by interacting with TRAF6 and recruiting RIP1, a key mediator of the NF-κB pathway. By regulating this interaction, p62 attenuates NF-κB activation, reducing proinflammatory cytokine production. Additionally, p62 intersects with the Nrf2–Keap1 axis by binding to Keap1 through its Keap1-interacting region (KIR), leading to the autophagic degradation of Keap1. This stabilizes Nrf2, promoting its nuclear translocation and transcriptional activation of antioxidant response element (ARE)-driven genes. The resulting upregulation of cytoprotective enzymes (e.g., HO-1 and NQO1) reduces oxidative stress and restores redox balance. Together, these pathways underscore p62 as a central node in the regulation of inflammation, oxidative stress, and cellular homeostasis in PD

Through the Nrf2/NF-ĸB/NLRP3 pathways, Nrf2-mediated NLRP3 suppression also occurs with the assistance of p62, a crucial regulator that links NF-ĸB signaling and autophagy [82]. P62 plays a dual role: it mediates NF-κB activation through its interaction with atypical protein kinase C (αPKC), facilitating the recruitment of TRAF6, which activates RIP1 [83]. IκB is then phosphorylated and degraded as a result of RIP1-activating IκB kinase (IKK), which allows NF-κB to translocate to the nucleus and transcribe proinflammatory genes [84]. Moreover, p62 promotes autophagy, a vital mechanism for degrading ubiquitinated NLRP3 inflammasome components, effectively reducing their activation, as shown in Fig. 3.

Autophagy also sustains Nrf2 activation by preventing its degradation via keap1, ensuring antioxidant and anti-inflammatory responses in PD [85].

### Additional Regulatory Mechanisms Mitigating NLRP3 in PD

Major histocompatibility complex (MHC) antigens and proinflammatory cytokines are produced by activated microglia, which also transform into phagocytic cells. Toll-like receptors 2 and 4 (TLR-2/4) and cyclo-oxygenase 2 (COX-2) regulate microglial activation, which is crucial for the neuroinflammatory response. Nrf2 activation attenuates oxidative stress by inducing anti-oxidant enzymes (e.g., HO-1, NQO1) which in turn reduce ROS levels. This redox balance suppresses NF-κB-driven transcription of pro-inflammatory mediators, including COX-2, TLR4 and TLR2 which are currently promoting anti-inflammatory cytokines such as IL-10. NRF2 can also influence inflammatory gene expression through epigenetic modulation, further sustaining a neuroprotective phenotype. Recently, Magnolol was shown to inhibit M1 polarization and induce M2 polarization via the Nrf2/HO-1/NLRP3 pathway [86]. Irisin was revealed to have neuroprotective effects on PD by activating the ERK, P38MAPK and AMPK pathways and then upregulating Nrf2, HO-1, and Bcl-2 independently of NF-κB, reducing the release of cytokines and the NLRP3 inflammasome and Caspase-1 [87]. Therefore, Nrf2 establishes distinct routes for controlling other elements of NLRP3 inflammasome-mediated damage to cells, including mitochondria, by interacting with several mediators [88]. Collectively, these interlinked mechanisms demonstrate that the Nrf2-Keap1 and NLRP3 pathways form a dynamic regulatory axis in PD, in which redox balance mitochondrial integrity and inflammatory signaling are tightly coupled.

## Redox-Dependent Modifications of Keap1 as Molecular Switch in PD

Keap1 interacts with several other redox-sensitive signaling pathways that modulate the activity of the NLRP3 inflammasome. Keap1 has been shown to modulate the activation of NF-κB via its impact on IkB kinase (IKK). By stabilizing IKK, Keap1 prevents NF-κB nuclear translocation, thereby reducing NLRP3 priming [89]. The interaction between Keap1 and thioredoxin (TRX) gives Nrf2–NLRP3 even more control. Thioredoxin-interacting protein (TXNIP) is usually linked to TRX, which stops TXNIP from turning on NLRP3. When oxidative stress is present, oxidized TRX releases TXNIP. This helps TXNIP bind to NLRP3 and start the inflammasome. The interaction between Keap1 and TRX may influence this process by altering TRX’s availability and redox state. Furthermore, through the TRX–TXNIP axis, Nrf2 activation indirectly inhibits NLRP3 activation by promoting the production of antioxidant enzymes that preserve TRX in a reduced state [90]. Apart from this redox axis Keap1 also works independently of Nrf2 to modify the mitogen-activated protein kinase (MAPK) pathway. Keap1 facilitates redox-sensitive regulation of kinase activation by serving as a scaffold for upstream MAPK regulators, including PGAM5, and Ikkβ Keap1’s function as a substrate adapter for cullin-3 E3 ligase enables it to target MAPK pathway components for ubiquitination and destruction, whereas oxidative alteration of its cysteine residue can change these protein–protein interactions [91]. These mechanisms affect the phosphorylation of stress-responsive kinases, including P-38 and JNK, which are both triggered by ROS and are essential for NLRP3 priming and activation of the downstream inflammatory genes. Furthermore, Keap1 collaborates with the chaperone proteins, heat shock proteins (HSPs) [92], and tripartite motif-containing proteins (Trims) [93] to regulate NLRP3 ubiquitination and degradation. Keap1 functions as a principal cytoplasmic repressor of Nrf2, and is sensitive to redox-dependent post-translational modifications (PTMs). In PD, mitochondrial ROS and electrophilic metabolites modify specific cysteine residues on Keap1, altering its ability to target Nrf2 for degradation. Owing to its reactive nature, many posttranslational changes [94] are shown in Table 2. They function via a different, noncanonical method of Nrf2 activation. Specifically, two proteins, ubiquitination and S-nitrosation, not only alter Keap1 residues but are also directly dependent on Keap1 and have been shown to affect its ability to regulate Nrf2 while simultaneously influencing the process mediated by NLRP3 [105]. Many oxidative electrophilic PTMs (e.g., cysteine oxidation at Cys151, Cys273 and Cys288) promote Nrf2 activation, leading to antioxidant gene induction, reduced ROS burden and limited TXNIP released from TRX thereby suppressing NLRP3 assembly. Others, such as phosphorylation and ubiquitination, influence MAPK-dependent NLRP3 priming independently of Nrf2. Through these mechanisms Keap1 PTMs act not only as a redox sensor but also as an upstream modulator of inflammatory signaling in PD. Collectively these PTMs serve as a molecular “redox switch” that senses oxidative imbalance, linking PD-associated oxidative stress directly to transcriptional antioxidant responses.

**Table 2:** Post-translation modification of keap1 and their relevance to NLRP3 regulation

Cysteine residue/site	Modification	Functional consequences	Impact on NLRP3 pathway	Reference
Cys151	Oxidation/alkylation	Disrupts Keap1-cullin-3 interaction, stabilizes Nrf2	ROS and limits TXNIP release from TRX and suppress NLRP3 assembly	[95, 96]
Cys273	S-nitrosation	Weakens Keap1–Nrf2 binding	Increase Nrf2-dependent antioxidant gene expression by reducing NLRP3 priming	[97], [98]
Multiple cysteines	Ubiquitination	Targets Nrf2 for degradation via Keap1	Nrf2 stabilization, reduces NF-κB- and MAPK-mediated NLRP3 priming	[99]
Cys257, Cys297	Glutathionylation	Modulates redox sensitivity and Nrf2 repression	Maintains TRX in a reduced state, prevents TXNIP-NLRP3 interaction	[100]
Multiple cysteines	Carbonylation	Irreversible oxidation, conformational instability	Skews redox balance toward anti-inflammatory state, limiting NLRP3 activation	[101]
Cys319	Sulfhydrylation	Enhance Nrf2 release under oxidative stress	Antioxidant effect reduces inflammasome activation	[102]
Multiple cysteines	Phosphorylation	Alter Keap1–Nrf2 interaction	Modulates MAPK-dependent NLRP3 priming	[103]
Multiple cysteines	Glycosylation	May alter protein folding and redox sensitivity	Indirectly affecting NLRP3 by reducing proinflammatory signaling	[104]

PTMs in Keap1 not only serve as a redox sensor for NRF 2 activation but also indirectly modulate NLRP 3 inflammation priming and activation via ROS production TRX-TXNIP axis modulation and MAPK signaling regulation. This dual regulation is particularly significant in PD where oxidative stress and chronic neuron inflammation are closely interconnected

## Experimental Outcomes of Combining Nrf2 Activators with the NLRP3 Inflammasome

Owing to their ability to produce significant levels of ROS as a metabolic byproduct through the metabolism of dopamine by MAO and autoxidation, dopaminergic neurons are susceptible to damage due to OS [106]. In fact, a number of studies have shown that PD patients’ blood and CSF contain lower amounts of antioxidants and higher indicators of oxidative damage, which are connected to the Nrf2 pathway [107]. One promising strategy for preserving cell homeostasis seems to be the activation of Nrf2, which fights OS [108]. Even if some of the mechanisms are still unclear, Nrf2 may be a good therapeutic target for PD. In amyloid precursor protein plus presenilin-1 (APP/PS1) mice, Nrf2 overexpression caused by antioxidant therapy has been demonstrated to improve neuronal apoptosis, increase thioredoxin-1 (TRX-1), and prevent the formation of NLRP3 inflammatory bodies [109]. Phase 2 clinical trials are currently being conducted on the use of EGCG to treat PD. The most effective medication for reducing PD symptoms is levodopa, sometimes known as L-DOPA. However, long-term L-DOPA use causes undesirable side effects and may increase the production of ROS [110]. By inhibiting microglial activation, polydatin (PLD) has been shown to eliminate the degeneration of motor neurons in the brains of LPS-induced mice. Furthermore, PLD treatment caused a significant increase in Nrf2 levels and prevented NF-κB activation in rats [111]. Chalcone-type chemicals, which include an α,β-unsaturated carbonyl group, change the cysteine residues of Keap1 via Michael addition, stimulating Nrf2 [112]. Nrf2 activator molecules with an α,β-unsaturated carbonyl group exhibit strong anti-inflammatory and antioxidant properties. Positive benefits against neurodegeneration have been demonstrated for molecules that can operate on inflammatory and oxidative damage systems [113]. Lee [114] demonstrated that isothiocyanate (ITC) derivatives inhibit inflammatory responses in dopaminergic neurons by causing the production of antioxidant Nrf2-dependent enzyme-encoding genes. Oral trehalose added to drinking water has been shown to increase autophagy, reduce damage to substantia nigra dopaminergic neurons, and promote the nuclear translocation of Nrf2 and the expression of downstream antioxidant enzymes [115]. Both DMF and MMF have neuroprotective effects and prevent neuronal toxicity [116]. Additionally, quercetin prevents the upregulation of NO and iNOS in PC12 cells and suppresses the production of proinflammatory genes (IL-1β, COX-2, and TNF-α) in zebrafish [117]. In MPTP-induced dopaminergic neurotoxicity in mice, two structural analogs of CDDO (TP-319 and TP-500), which are produced by the cyclization of squalene, have been shown to increase blood–brain barrier permeability and provide protection against inflammation and oxidative stress [118]. By downregulating NF-κB and increasing Nrf2 activation, the entkauranoid diterpenoid glaucocalyxin-B, which is produced from Rabdosia japonica, was demonstrated to decrease oxidative stress and inflammation in a PD rat model caused by LPS [119]. Virtual screening of the Asinex and Chemdiv databases revealed KKPA4026, another strong Nrf2 activator. In response to KKPA4026, BV2 cells express the Nrf2-dependent antioxidant enzymes HO-1, glutamate-cysteine ligase catalytic subunit, glutamate-cysteine ligase regulatory subunit, and NAD (P)H:quinone oxidoreductase 1 [120]. Similarly, in a model of PD caused by MPTP, ALGERNON2 reduced the release of proinflammatory cytokines and stabilized the cyclin D1/p21 complex by inhibiting Dyrk1A activity, leading to Nrf2-dependent antioxidant and anti-inflammatory responses [121]. Cryptotanshinone, a monomer, can reduce LPS-induced neuroinflammation in BV-2 microglia via the Nrf2/HO-1 signaling pathway [122]. The experimental outcomes and preclinical studies showing the impact of Nrf2 on the NLRP3 inflammasome are shown in Table 1.

## Nrf2/HO-1 Signaling Downregulation Promotes Mitochondrial Dysfunction and Neuroinflammation

Nrf2/HO-1 has been shown to be a significant pathway involved in suppressing oxidative stress and NLRP3-induced neuroinflammation, but downregulation of this pathway plays a critical role in exacerbating neurodegeneration. Mitochondria are producers of ROS during oxidative phosphorylation. Nrf2 is the master regulator of antioxidant defense, and impaired Nrf2 activation leads to mitochondrial damage [123]. This mitochondrial damage amplifies ROS production, fuelling neuroinflammation by activating microglia, increasing NF-κB signaling and increasing NLRP3 activation. Nuclear factor-κB (NF-κB) and Nrf2 are two important transcription factors that can be very important in regulating oxidative stress and triggering detoxifying enzymes [124]. According to preclinical research, neuronal ATP levels and mitochondrial respiration activity are decreased when the Nrf2 gene is knocked out [125]. A study showed that when Nrf2 is absent in mice, astrocyte activation increases, and NF-κB signaling pathways are modulated, causing nerve damage and an inflammatory response [126]. The transcription factor NF-κB comprises c-rel, p50, p52, RelA (p65), and RelB. NF-κB is activated by bacterial lipopolysaccharide (LPS), a component of gram-negative bacteria, interleukin (IL-1β), and tumor necrosis factor (TNF-α). Because Nrf2 suppresses NF-κB and downstream proinflammatory cytokines, its absence may result in more severe inflammation in astrocytes [127]. The simultaneous activation of NF-κB and Nrf2 creates antagonistic interactions where p65, a subunit of NF-κB, suppresses Nrf2 activity to drive the expression of ARE genes, such as HO-1, without p65’s own transcriptional activity. This occurs through mechanisms such as competitive interference, where p65 competes with Nrf2 for coactivators (e.g., CBP or P300) and disrupts the Nrf2 transcriptional complex at the ARE. Interference between p65 and Nrf2 diminishes the ability of cells to neutralize ROS and cause oxidative damage [128]. Inflammation is increased by downregulating Nrf2 and NF-κB activation in inflammatory cells, such as T cells, microglia/macrophages, and astrocytes. Although studies have shown that Nrf2 and Nrf2-activating chemicals have opposing effects on the NLRP3 inflammasome that are not dependent on Nrf2-mediated gene expression, both effects are independent of the transcriptional activity of Nrf2 [129].

## Therapeutic implications

### Nrf2 Activators and NLRP3 Inhibitors as Therapeutic Agents in PD

Nrf2 activators and NLRP 3 inhibitors have shown encouraging neuroprotective effects in preclinical models of PD; translation into clinical benefits remains challenging. Many Nrf2 inducers including electrophilic agents such as dimethyl fumarate and natural products like sulforaphane, act on multiple cellular targets beyond Keap1, raising the possibility of off-target effects such as immunomodulation, altered drug metabolism or unintended transcription changes [130]. Similar to this, NLRP3 inhibitors may weaken the host’s immune system, especially during an infection, even though they are efficient at preventing inflammasome activation [131]. Another significant obstacle is the permeability of the blood–brain barrier, as many small compounds that demonstrate strong in vitro efficacy have limited exposure to the central nervous system (CNS) in vivo, necessitating high doses that may increase systemic toxicity. Additionally, the results of clinical studies of Nrf2 activators in neurodegenerative diseases have been inconsistent, most likely because of genetic differences between individuals (e.g., NLRP3 or NFE2L2), the stage of the disease at intervention, and the differences between the complex pathology of human PD and toxin-based animal models. Consequently, the development of agents with high CNS bioavailability, selective target engagement, and minimal adverse effects, confirmed by well-powered, stage-stratified clinical trials, will therefore be necessary to determine their clinical utility, even though inducers and NLRP3 inhibitors continue to be promising candidates for disease-modifying therapy. Tables 3 and Table 4 depict the Nrf2 activators and NLRP3 inhibitors acknowledging BBB penetrations, off-target effects and limitations. The current ongoing trial of Nrf2 activator is shown in Table 5.

**Table 3:** Nrf2 inducers as therapeutic agents

Nrf2 activator	Mechanism of action	BBB permeability	Off-target effects	Limitation	Reference
Sulforaphane	Isothiocyanate group covalently modifies Keap1 thiol and Nrf2 translocation	Good BBB penetration in rodents but human delivery is not fully quantified	May modulate drug-metabolizing enzymes	Poor human pharmacokinetic data, uncleared long safety data in elderly PD patients	[132]
Vitamin D_3_	Activates Nrf2 indirectly via vitamin D receptor, enhances antioxidant expression	Lipophilic, cross BBB but CNS levels may reflect peripheral vitamin D status	Hypercalcemia with renal calculi risk with chronic high-dose use	Limited PD specific RCTs; effects may be secondary to systemic anti-inflammatory and metabolic benefits	[133]
Resveratrol	Polyphenol that activates Nrf2 via the AMPK/SIRT pathway, enhances mitochondrial biogenesis	Cross BBB but rapidly metabolized	Potential estrogenic effect, drug-drug interaction	Poor bioavailability, inconsistent efficacy in human studies	[134]
EGCG	Indirect Nrf2 activation via kinase signaling modulation	Limited BBB penetration, improved with lipid-based carriers	Possible hepatotoxicity at high dose	Limited PD specific evidence, hepatoxicity risk at high dose	[135]
Pioglitazone	PPARγ agonist indirectly activates Nrf2	Lipophilic, cross BBB but CNS levels may vary with dosing	Weight gain, fluid retention, possible cardiovascular risk	Mixed efficacy in PD human studies; safety concerns limit long-term high-dose use	[136]

**Table 4:** Ongoing clinical trials of Nrf2 activators

Nrf2 activator	Topic	Chemical formula	phase	Registration no	Reference
Pentoxifylline	Clinical study to compare safety and efficacy of pentoxifylline having PD	C_13_H_18_O_11_	Recruiting	NCT05962957	[137]

**Table 5:** NLRP3 inhibitors as therapeutic agents

NLRP3 inhibitor	Mechanism of action	BBB permeability	Off-target effect	limitations	Reference
NodThera’s NT-0796	Brain-penetrant, small molecule NLRP3 inhibitor	Designed for BBB penetration, promising CNS exposure	Safety profile still under investigation	Still in early phase trial, limited data	[138]
VTX3232	Highly selective, oral bioavailable NLRP3 inhibitor	BBB penetration profile yet not established	Possible immune suppression	Further research is needed to confirm efficacy	[139]
Senloflast	Investigational CNS-penetrant NLRP3 inhibitor	Reported BBB penetration in animal model	Pre-clinical evidence for microglial inflammasome suppression	No PD-specific in vivo data; clinical proof-of-concept pending	[140]
OLT Dapansutrile	Orally active NLRP3 inhibitor, blocking ATPase activity	Partial BBB penetration	GI symptoms, rare hepatoxicity	No PD-specific human data, CNS target analogue may be required	[141]
ZIYL 1	Novel small-molecule NLRP3 inhibitor with reported CNS activity	Designed for BBB penetration but requires confirmation in vivo	Mechanistic off-target effects unknown	In very early development stage, require further confirmation	[142]
RRX001	Epigenetic modulator and NLRP3 inhibitor via ROS signaling and NF-κB suppression	Likely BBB penetration due to lipophilicity	May affect multipole transcriptional regulators; unknown CNS safety	Lead to infusion-related reactions like venous inflammation and pain; safety PD-specificity needs further investigation	[143]

## Conclusion

For several years, the influence of the immune system, particularly that of the glial system, has been emphasized as central to PD pathogenesis. Microglia and astrocytes contribute to neuroinflammation through multiple mechanisms, with the Nrf2-NLRP3 axis emerging as a key link between oxidative stress, mitochondrial dysfunction, and inflammation. This axis not only shapes neuroinflammation but also affects mitochondrial integrity. Its importance to PD is supported by multiple in vitro and in vivo studies, including α-synuclein aggregation and mitochondrial damage. Impaired Nrf2 signaling permits overactivation, while inflammasome hyperactivity can suppress Nrf2, forming a self-perpetuating cycle of oxidative stress and inflammation. Failures in mitochondrial quality control and ferroptosis appear to drive this imbalance, highlighting the therapeutic value of restoring antioxidant defense while limiting inflammasome activation. Nrf2 inducers have demonstrated efficacy in preclinical PD models partly by preventing NLRP3 activation. However, clinical translation remains limited by off-target effects, blood–brain barrier challenges and variability in pathway regulation. The need to precisely develop targeted therapeutic approaches using Nrf2 inducers grows as new research and developments deepen our understanding of the NLRP3/Nrf2 interaction. To achieve this, more extensive and multidisciplinary research initiatives are needed. However, this study will expand our understanding of how the NLRP3 inflammasome is regulated and the diseases associated with it.

## Data Availability

No datasets were generated or analysed during the current study.

## Abbreviations

PD: Parkinson disease
NRF2: Nuclear factor erythroid-2 related factor 2
keap-1: Kelch-like ECH-associated protein 1
NLRP3: NLR family pyrin domain containing protein 3
ROS: Reactive oxygen species
HO-1: Heme oxygenase 1
TXNIP: Thioredoxin interacting protein
NF-κB: Nuclear factor kappa B
IL: Interleukin
NQO1: NADPH quinone oxidoreductase-1
GPx: Glutathione peroxidase
CBP: CREB binding protein
IKKβ: Inhibitory kappa B kinaseβ
ARE: Antioxidant response element
TRAF6: Tumor necrosis factor receptor associated factor 6
RIP1: Receptor interacting protein
P-62/(SQSTM1): Sequestosome 1
CARD: Caspase recruitment domain
CK1: Cysteine kinase 2
MPTP: 1-Methyl-4-phenyl-1,2,3,6-tetrahydropyridine
LPS: Lipopolysaccharide

## References

[1] GBD 2016 Neurology Collaborators. Global, regional, and national burden of neurological disorders, 1990–2016: a systematic analysis for the Global Burden of Disease Study 2016. Lancet Neurol. 2019;18 (5):459–480. 10.1016/S1474-4422 (18)30499-X. 10.1016/S1474-4422(18)30499-X PMC6459001 30879893

[2] Dorsey ER, Elbaz A, Nichols E, Abd-Allah F, Abdelalim A, Adsuar JC et al (2018) Global, regional, and national burden of Parkinson’s disease, 1990–2016: a systematic analysis for the Global burden of disease study 2016. Lancet Neurol 17(11):939–953. 10.1016/S1474-4422(18)30295-3 30287051 10.1016/S1474-4422(18)30295-3 PMC6191528

[3] Absalyamova M, Traktirov D, Burdinskaya V, Artemova V, Muruzheva Z, Karpenko M (2025) Molecular basis of the development of Parkinson’s disease. Neuroscience 26(565):292–300 10.1016/j.neuroscience.2024.12.009 39653246

[4] Chakrabarti S, Bisaglia M (2023) Oxidative stress and neuroinflammation in Parkinson’s disease: the role of dopamine oxidation products. Antioxidants 12(4):955 37107329 10.3390/antiox12040955 PMC10135711

[5] Guo M, Wang J, Zhao Y, Feng Y, Han S, Dong Q et al (2020) Microglial exosomes facilitate α-synuclein transmission in Parkinson’s disease. Brain 143(5):1476–1497 32355963 10.1093/brain/awaa090 PMC7241957

[6] Saha S, Buttari B, Panieri E, Profumo E, Saso L (2020) An overview of Nrf2 signaling pathway and its role in inflammation. Molecules 25(22):5474 33238435 10.3390/molecules25225474 PMC7700122

[7] Suzuki T, Takahashi J, Yamamoto M (2023) Molecular basis of the KEAP1-NRF2 signaling pathway. Mol Cells 46(3):133–141 36994473 10.14348/molcells.2023.0028 PMC10070164

[8] Zgorzynska E, Dziedzic B, Walczewska A (2021) An overview of the Nrf2/ARE pathway and its role in neurodegenerative diseases. Int J Mol Sci 22(17):9592 34502501 10.3390/ijms22179592 PMC8431732

[9] Yu J, Zhao Z, Li Y, Chen J, Huang N, Luo Y (2024) Role of NLRP3 in Parkinson’s disease: specific activation especially in dopaminergic neurons. Heliyon 10(7) 38596076 10.1016/j.heliyon.2024.e28838 PMC11002585

[10] Ma Q (2023) Pharmacological inhibition of the NLRP3 inflammasome: structure, molecular activation, and inhibitor-NLRP3 interaction. Pharmacol Rev 75(3):487–520 36669831 10.1124/pharmrev.122.000629 PMC10121800

[11] de Araújo FM, Cuenca-Bermejo L, Fernández-Villalba E, Costa SL, Silva VDA, Herrero MT (2022) Role of microgliosis and NLRP3 inflammasome in Parkinson’s disease pathogenesis and therapy. Cell Mol Neurobiol 42(5):1283–1300 33387119 10.1007/s10571-020-01027-6 PMC11421755

[12] Yang Q, Zhou J (2019) Neuroinflammation in the central nervous system: symphony of glial cells. Glia 67(6):1017–1035 30548343 10.1002/glia.23571

[13] Darvish Khadem M, Tabandeh MR, Haschemi A, Kheirollah A, Shahriari A (2022) Dimethyl itaconate reprograms neurotoxic to neuroprotective primary astrocytes through the regulation of NLRP3 inflammasome and NRF2/HO-1 pathways. Mol Cell Neurosci 1(122) 10.1016/j.mcn.2022.103758 35868484

[14] Shi X, Zhou H, Wei J, Mo W, Li Q, Lv X (2022) The signaling pathways and therapeutic potential of itaconate to alleviate inflammation and oxidative stress in inflammatory diseases. Redox Biol 1 10.1016/j.redox.2022.102553 PMC9713374 36459716

[15] Huang J, Zhang X, Yang X, Yv Q, Ye F, Chen S et al (2024) Baicalin exerts neuroprotective actions by regulating the Nrf2-NLRP3 axis in toxin-induced models of Parkinson’s disease. Chem Biol Interact 5(387) 10.1016/j.cbi.2023.110820 38016618

[16] Suzuki T, Yamamoto M (2015) Molecular basis of the Keap1-Nrf2 system. Free Radic Biol Med 88:93–100 26117331 10.1016/j.freeradbiomed.2015.06.006

[17] Madden SK, Itzhaki LS. Structural and mechanistic insights into the Keap1-Nrf2 system as a route to drug discovery. Biochimica et Biophysica Acta (BBA) - Proteins and Proteomics. 2020 Jul 1;1868 (7):140405. 10.1016/j.bbapap.2020.140405 32120017

[18] Bento-Pereira C, Dinkova-Kostova AT, John Wiley and Sons Inc (2021) Activation of transcription factor Nrf2 to counteract mitochondrial dysfunction in Parkinson’s disease. Med Res Rev 41:785–802 32681666 10.1002/med.21714

[19] Yang X xing, Yang R, Zhang F. Role of Nrf2 in Parkinson’s disease: toward new perspectives. Front Pharmacol. 2022 Jun 24;13. 10.3389/fphar.2022.919233 PMC9263373 35814229

[20] Heurtaux T, Bouvier DS, Benani A, Helgueta Romero S, Frauenknecht KBM, Mittelbronn M, et al. Normal and pathological NRF2 signalling in the central nervous system. Vol. 11, Antioxidants. MDPI; 2022. 10.3390/antiox11081426 PMC9394413 35892629

[21] Saito R, Suzuki T, Hiramoto K, Asami S, Naganuma E, Suda H et al (2016) Characterizations of three major cysteine sensors of Keap1 in stress response. Mol Cell Biol 36(2):271–284 26527616 10.1128/MCB.00868-15 PMC4719294

[22] Liu T, Lv YF, Zhao JL, You QD, Jiang ZY (2021) Regulation of Nrf2 by phosphorylation: consequences for biological function and therapeutic implications. Free Radic Biol Med 20(168):129–141 10.1016/j.freeradbiomed.2021.03.034 33794311

[23] Uddin MS, Mamun A Al, Jakaria M, Thangapandiyan S, Ahmad J, Rahman MA, et al. Emerging promise of sulforaphane-mediated Nrf2 signaling cascade against neurological disorders. Science of the Total Environment. 2020 Mar 10;707:135624. 10.1016/j.scitotenv.2019.135624 31784171

[24] Liu S, Pi J, Zhang Q (2022) Signal amplification in the KEAP1-NRF2-ARE antioxidant response pathway. Redox Biol 1:54 10.1016/j.redox.2022.102389 PMC9287733 35792437

[25] Upadhayay S, Mehan S (2021) Targeting Nrf2/HO-1 anti-oxidant signaling pathway in the progression of multiple sclerosis and influences on neurological dysfunctions. Brain Disord 3(1)

[26] Huang HC, Nguyen T, Pickett CB (2002) Phosphorylation of Nrf2 at Ser-40 by protein kinase C regulates antioxidant response element-mediated transcription. J Biol Chem 277(45):42769–42774 12198130 10.1074/jbc.M206911200

[27] Petsouki E, Cabrera SNS, Heiss EH (2022) AMPK and NRF2: interactive players in the same team for cellular homeostasis? Free Radic Biol Med 1(190):75–93 10.1016/j.freeradbiomed.2022.07.014 35918013

[28] Villavicencio Tejo F, Quintanilla RA (2021) Contribution of the Nrf2 pathway on oxidative damage and mitochondrial failure in Parkinson and Alzheimer’s disease. Antioxidants 10(7):1069 34356302 10.3390/antiox10071069 PMC8301100

[29] Sun Y, He L, Wang T, Hua W, Qin H, Wang J et al (2020) Activation of p62-Keap1-Nrf2 pathway protects 6-hydroxydopamine-induced ferroptosis in dopaminergic cells. Mol Neurobiol 57(11):4628–4641 32770451 10.1007/s12035-020-02049-3

[30] Komatsu M, Kurokawa H, Waguri S, Taguchi K, Kobayashi A, Ichimura Y, Sou YS, Ueno I et al (2010) The selective autophagy substrate p62 activates the stress responsive transcription factor Nrf2 through inactivation of Keap1. Nat Cell Biol 12(3):213–223. 10.1038/ncb2021 20173742 10.1038/ncb2021

[31] Soares MP, Seldon MP, Gregoire IP, Vassilevskaia T, Berberat PO, Yu J et al (2004) Heme oxygenase-1 modulates the expression of adhesion molecules associated with endothelial cell activation. J Immunol 172(6):3553–3563 15004156 10.4049/jimmunol.172.6.3553

[32] Liu X, Zhang X, Ding Y, Zhou W, Tao L, Lu P et al (2017) Nuclear factor E2-related factor-2 negatively regulates NLRP3 inflammasome activity by inhibiting reactive oxygen species-induced NLRP3 priming. Antioxid Redox Signal 26(1):28–43 27308893 10.1089/ars.2015.6615 PMC5198158

[33] Yu X, Matico RE, Miller R, Chauhan D, Van Schoubroeck B, Grauwen K et al (2024) Structural basis for the oligomerization-facilitated NLRP3 activation. Nat Commun 15(1):1164 38326375 10.1038/s41467-024-45396-8 PMC10850481

[34] Paik S, Kim JK, Silwal P, Sasakawa C, Jo EK (2021) An update on the regulatory mechanisms of NLRP3 inflammasome activation. Cell Mol Immunol 18(5):1141–1160 33850310 10.1038/s41423-021-00670-3 PMC8093260

[35] Pike AF, Szabò I, Veerhuis R, Bubacco L (2022) The potential convergence of NLRP3 inflammasome, potassium, and dopamine mechanisms in Parkinson’s disease. NPJ Parkinsons Dis 8(1):32 35332154 10.1038/s41531-022-00293-z PMC8948240

[36] Jo EK, Kim JK, Shin DM, Sasakawa C (2016) Molecular mechanisms regulating NLRP3 inflammasome activation. Cell Mol Immunol 13(2):148–159 26549800 10.1038/cmi.2015.95 PMC4786634

[37] Xu J, Núñez G. The NLRP3 inflammasome: activation and regulation. 2022. 10.1016/j.tibs.2022.10.002 PMC10023278 36336552

[38] Temiz-Resitoglu M, Kucukkavruk SP, Guden DS, Cecen P, Sari AN, Tunctan B et al (2017) Activation of mTOR/IκB-α/NF-κB pathway contributes to LPS-induced hypotension and inflammation in rats. Eur J Pharmacol 802:7–19 28228357 10.1016/j.ejphar.2017.02.034

[39] Niu T, De Rosny C, Chautard S, Rey A, Patoli D, Groslambert M et al (2021) NLRP3 phosphorylation in its LRR domain critically regulates inflammasome assembly. Nat Commun 12(1):5862 34615873 10.1038/s41467-021-26142-w PMC8494922

[40] Barry R, John SW, Liccardi G, Tenev T, Jaco I, Chen CH et al (2018) SUMO-mediated regulation of NLRP3 modulates inflammasome activity. Nat Commun 9(1):3001 30069026 10.1038/s41467-018-05321-2 PMC6070540

[41] Cao G, Jiang N, Hu Y, Zhang Y, Wang G, Yin M et al (2016) Ruscogenin attenuates cerebral ischemia-induced blood-brain barrier dysfunction by suppressing TXNIP/NLRP3 inflammasome activation and the MAPK pathway. Int J Mol Sci 17(9):1418 27589720 10.3390/ijms17091418 PMC5037697

[42] Zhao W, Ma L, Cai C, Gong X (2019) Caffeine inhibits NLRP3 inflammasome activation by suppressing MAPK/NF-κB and A2ar signaling in LPS-induced THP-1 macrophages. Int J Biol Sci 15(8):1571–1581 31360100 10.7150/ijbs.34211 PMC6643212

[43] Abderrazak A, Syrovets T, Couchie D, El Hadri K, Friguet B, Simmet T, Elsevier B.V. et al (2015) NLRP3 inflammasome: from a danger signal sensor to a regulatory node of oxidative stress and inflammatory diseases. Redox Biol 4:296–307 10.1016/j.redox.2015.01.008 PMC4315937 25625584

[44] Wang Z, Zhang S, Xiao Y, Zhang W, Wu S, Qin T et al (2020) NLRP3 inflammasome and inflammatory diseases. Oxid Med Cell Longev. 10.1155/2020/4063562 32148650 10.1155/2020/4063562 PMC7049400

[45] Zhou Y, Lu M, Du RH, Qiao C, Jiang CY, Zhang KZ et al (2016) MicroRNA-7 targets Nod-like receptor protein 3 inflammasome to modulate neuroinflammation in the pathogenesis of Parkinson’s disease. Mol Neurodegener 11(1):28 27084336 10.1186/s13024-016-0094-3 PMC4833896

[46] Li S, Liang X, Ma L, Shen L, Li T, Zheng L, et al. MiR-22 sustains NLRP3 expression and attenuates H. pylori-induced gastric carcinogenesis. Oncogene. 2018 Feb 23;37 (7):884–96. 10.1038/onc.2017.381 29059152

[47] Li D, Yang H, Ma J, Luo S, Chen S, Gu Q (2018) MicroRNA-30e regulates neuroinflammation in MPTP model of Parkinson’s disease by targeting Nlrp3. Hum Cell 31(2):106–115 29274035 10.1007/s13577-017-0187-5 PMC5852205

[48] Xiao L, Jiang L, Hu Q, Li Y (2017) Microrna-133b ameliorates allergic inflammation and symptom in murine model of allergic rhinitis by targeting Nlrp3. Cell Physiol Biochem 42(3):901–912 28662502 10.1159/000478645

[49] Long F, Kou C, Li K, Wu J, Wang Q (2020) MiR-223-3p inhibits rTp17-induced inflammasome activation and pyroptosis by targeting NLRP3. J Cell Mol Med 24(24):14405–14414 33145937 10.1111/jcmm.16061 PMC7754033

[50] Das B, Sarkar C, Rawat VS, Kalita D, Deka S, Agnihotri A (2021) Promise of the NLRP3 inflammasome inhibitors in in vivo disease models. Molecules 26(16):4996 34443594 10.3390/molecules26164996 PMC8399941

[51] Juliana C, Fernandes-Alnemri T, Wu J, Datta P, Solorzano L, Yu JW et al (2010) Anti-inflammatory compounds parthenolide and bay 11–7082 are direct inhibitors of the inflammasome. J Biol Chem 285(13):9792–9802 20093358 10.1074/jbc.M109.082305 PMC2843228

[52] Raneros AB, Bernet CR, Flórez AB, Suarez-Alvarez B (2021) An epigenetic insight into NLRP3 inflammasome activation in inflammation-related processes. Biomedicines 9(11):1614 34829842 10.3390/biomedicines9111614 PMC8615487

[53] Sweeney MD, Sagare AP, Zlokovic BV (2018) Blood–brain barrier breakdown in Alzheimer disease and other neurodegenerative disorders. Nat Rev Neurol 14(3):133–150. 10.1038/nrneurol.2017.188 29377008 10.1038/nrneurol.2017.188 PMC5829048

[54] Gray MT, Woulfe JM (2015) Striatal blood–brain barrier permeability in Parkinson’s disease. J Cereb Blood Flow Metab 35(5):747–750. 10.1038/jcbfm.2015.32 25757748 10.1038/jcbfm.2015.32 PMC4420870

[55] Erickson MA, Banks WA (2013) Blood–brain barrier dysfunction as a cause and consequence of Alzheimer’s disease. J Cereb Blood Flow Metab 33(10):1500–1513. 10.1038/jcbfm.2013.135 23921899 10.1038/jcbfm.2013.135 PMC3790938

[56] Trudler D, Nazor KL, Eisele YS, Grabauskas T, Dolatabadi N, Parker J, et al. Soluble α-synuclein–antibody complexes activate the NLRP3 inflammasome in hiPSC-derived microglia. Proceedings of the National Academy of Sciences. 2021 Apr 13;118 (15). 10.1073/pnas.2025847118 PMC8054017 33833060

[57] Role of NLRP3 inflammasome in Parkinson’s disease and therapeutic considerations. 10.3233/JPD-223290 PMC9661339 35988226

[58] Mechanisms of ferroptosis and emerging links to the pathology of neurodegenerative diseases. 10.3389/fnagi.2022.904152 PMC9273851 35837484

[59] Biasizzo M, Kopitar-Jerala N (2020) Interplay between NLRP3 inflammasome and autophagy. Front Immunol 9 10.3389/fimmu.2020.591803 PMC7583715 33163006

[60] Pike AF, Varanita T, Herrebout MAC, Plug BC, Kole J, Musters RJP et al (2021) α-synuclein evokes NLRP3 inflammasome-mediated IL-1β secretion from primary human microglia. Glia 69(6):1413–1428 33506583 10.1002/glia.23970 PMC8247862

[61] Li Y, Xia Y, Yin S, Wan F, Hu J, Kou L et al (2021) Targeting microglial α-synuclein/TLRs/NF-kappaB/NLRP3 inflammasome axis in Parkinson’s disease. Front Immunol 8:12 10.3389/fimmu.2021.719807 PMC8531525 34691027

[62] Dopamine signaling modulates microglial NLRP3 inflammasome activation: implications for Parkinson’s disease. 10.1186/s12974-022-02410-4 PMC8848816 35172843

[63] Role of microgliosis and NLRP3 inflammasome in Parkinson’s disease pathogenesis and therapy. 10.1007/s10571-020-01027-6 PMC11421755 33387119

[64] Park JE, Leem YH, Park JS, Kim SE, Kim HS (2023) Astrocytic Nrf2 Mediates the neuroprotective and anti-inflammatory effects of Nootkatone in an MPTP-induced Parkinson’s disease mouse model. Antioxidants 12(11):1999 38001852 10.3390/antiox12111999 PMC10669233

[65] Zgorzynska E, Dziedzic B, Walczewska A, MDPI (2021) An overview of the Nrf2/ARE pathway and its role in neurodegenerative diseases. Int J Mol Sci. 10.3390/ijms22179592 34502501 10.3390/ijms22179592 PMC8431732

[66] Haines DD, Tosaki A (2020) Heme degradation in pathophysiology of and countermeasures to inflammation-associated disease. Int J Mol Sci 21(24):9698 33353225 10.3390/ijms21249698 PMC7766613

[67] Jhang JJ, Yen GC, Chinese Soc Immunology (2017) The role of Nrf2 in NLRP3 inflammasome activation. Cell Mol Immunol 14:1011–1012 29129911 10.1038/cmi.2017.114 PMC5719138

[68] Tastan B, Arioz BI, Genc S (2022) Targeting NLRP3 inflammasome with Nrf2 inducers in central nervous system disorders. Front Immunol. 10.3389/fimmu.2022.865772 35418995 10.3389/fimmu.2022.865772 PMC8995746

[69] Teleanu DM, Niculescu AG, Lungu II, Radu CI, Vladâcenco O, Roza E, MDPI et al (2022) An overview of oxidative stress, neuroinflammation and neurodegenerative diseases. Int J Mol Sci. 10.3390/ijms23115938 10.3390/ijms23115938 PMC9180653 35682615

[70] Rui W, Li S, Xiao H, Xiao M, Shi J (2020) Baicalein attenuates neuroinflammation by inhibiting NLRP3/caspase-1/GSDMD pathway in MPTP-induced mice model of Parkinson’s disease. Int J Neuropsychopharmacol 23(11):762–773 32761175 10.1093/ijnp/pyaa060 PMC7745250

[71] Abu-Elfotuh K, Hamdan AME, Mohammed AA, Atwa AM, Kozman MR, Ibrahim AM et al (2022) Neuroprotective effects of some nutraceuticals against manganese-induced Parkinson’s disease in rats: possible modulatory effects on TLR4/NLRP3/NF-κB, GSK-3β, Nrf2/HO-1, and apoptotic pathways. Pharmaceuticals 15(12):1554 36559006 10.3390/ph15121554 PMC9785377

[72] Lu R, Zhou X, Zhang L, Hao M, Yang X (2024) Nrf2 deficiency exacerbates Parkinson’s disease by aggravating NLRP3 inflammasome activation in MPTP-induced mouse models and LPS-induced BV2 cells. J Inflamm Res 17:6277–6295 39281779 10.2147/JIR.S478683 PMC11401530

[73] Foresti R, Bains SK, Pitchumony TS, De Castro Brás LE, Drago F, Dubois-Randé JL et al (2013) Small molecule activators of the Nrf2-HO-1 antioxidant axis modulate heme metabolism and inflammation in BV2 microglia cells. Pharmacol Res 1(76):132–148 10.1016/j.phrs.2013.07.010 23942037

[74] Yang C, Mo Y, Xu E, Wen H, Wei R, Li S et al (2019) Astragaloside IV ameliorates motor deficits and dopaminergic neuron degeneration via inhibiting neuroinflammation and oxidative stress in a Parkinson’s disease mouse model. Int Immunopharmacol 1:75 10.1016/j.intimp.2019.05.036 31401385

[75] Palomino-Antolín A, Decouty-Pérez C, Farré-Alins V, Narros-Fernández P, Lopez-Rodriguez AB, Álvarez-Rubal M, et al. Redox regulation of microglial inflammatory response: fine control of NLRP3 inflammasome through Nrf2 and NOX4. Antioxidants. 2023 Sep 1;12 (9). 10.3390/antiox12091729 PMC10525647 37760032

[76] Yang W, Chen YH, Liu H, Qu HD (2015) Neuroprotective effects of piperine on the 1-methyl-4-phenyl-1,2,3,6-tetrahydropyridine-induced Parkinson’s disease mouse model. Int J Mol Med 36(5):1369–1376 26648012 10.3892/ijmm.2015.2356

[77] Yang S, Zhang X, Zhang H, Lin X, Chen X, Zhang Y et al (2021) Dimethyl itaconate inhibits LPS-induced microglia inflammation and inflammasome-mediated pyroptosis via inducing autophagy and regulating the Nrf-2/HO-1 signaling pathway. Mol Med Rep 24(3):672 34296312 10.3892/mmr.2021.12311 PMC8335742

[78] Shah S, Pushpa Tryphena K, Singh G, Kulkarni A, Pinjala P, Kumar KD (2024) Neuroprotective role of carvacrol via Nrf2/HO-1/NLRP3 axis in rotenone-induced PD mice model. Brain Res 1(1836) 10.1016/j.brainres.2024.148954 38649135

[79] Zhang C, Zhao M, Wang B, Su Z, Guo B, Qin L et al (2021) The Nrf2-NLRP3-caspase-1 axis mediates the neuroprotective effects of celastrol in Parkinson’s disease. Redox Biol 1:47 10.1016/j.redox.2021.102134 PMC8487081 34600334

[80] Li J, Yu J, Guo J, Liu J, Wan G, Wei X et al (2023) *Nardostachys jatamansi* and levodopa combination alleviates Parkinson’s disease symptoms in rats through activation of Nrf2 and inhibition of NLRP3 signaling pathways. Pharm Biol 61(1):1175–1185 37559448 10.1080/13880209.2023.2244176 PMC10416743

[81] Zhang C, Zhou J, Zhuo L, Zhang W, Lv L, Zhu L et al (2024) The TLR4/NF-κB/NLRP3 and Nrf2/HO-1 pathways mediate the neuroprotective effects of alkaloids extracted from *Uncaria rhynchophylla* in Parkinson’s disease. J Ethnopharmacol 28(333) 10.1016/j.jep.2024.118391 38797377

[82] Biasizzo M, Kopitar-Jerala N (2020) Interplay between NLRP3 inflammasome and autophagy. Front Immunol 9 10.3389/fimmu.2020.591803 PMC7583715 33163006

[83] Hennig P, Garstkiewicz M, Grossi S, Di Filippo M, French L, Beer HD (2018) The crosstalk between Nrf2 and inflammasomes. Int J Mol Sci 19(2):562 29438305 10.3390/ijms19020562 PMC5855784

[84] Diaz-Meco MT, Moscat J (2012) The atypical PKCs in inflammation: NF-κB and beyond. Immunol Rev 246(1):154–167 22435553 10.1111/j.1600-065X.2012.01093.x PMC3531713

[85] Han X, Sun S, Sun Y, Song Q, Zhu J, Song N et al (2019) Small molecule-driven NLRP3 inflammation inhibition via interplay between ubiquitination and autophagy: implications for Parkinson disease. Autophagy 15(11):1860–1881 30966861 10.1080/15548627.2019.1596481 PMC6844502

[86] Tao W, Hu Y, Chen Z, Dai Y, Hu Y, Qi M (2021) Magnolol attenuates depressive-like behaviors by polarizing microglia towards the M2 phenotype through the regulation of Nrf2/HO-1/NLRP3 signaling pathway. Phytomedicine 1(91) 10.1016/j.phymed.2021.153692 34411834

[87] Qiu R, Sun W, Su Y, Sun Z, Fan K, Liang Y et al (2024) Irisin’s emerging role in Parkinson’s disease research: a review from molecular mechanisms to therapeutic prospects. Life Sci 15(357) 10.1016/j.lfs.2024.123088 39357796

[88] Rajan S, Tryphena KP, Khan S, Vora L, Srivastava S, Singh SB, et al. Understanding the involvement of innate immunity and the Nrf2-NLRP3 axis on mitochondrial health in Parkinson’s disease. Vol. 87, Ageing research reviews. Elsevier Ireland Ltd; 2023. 10.1016/j.arr.2023.101915 36963313

[89] Yamamoto M, Kensler TW, Motohashi H (2018) The KEAP1-NRF2 system: a thiol-based sensor-effector apparatus for maintaining redox homeostasis. Physiol Rev 98(3):1169–1203 29717933 10.1152/physrev.00023.2017 PMC9762786

[90] Lv H, Zhu C, Wei W, Lv X, Yu Q, Deng X et al (2020) Enhanced Keap1-Nrf2/Trx-1 axis by daphnetin protects against oxidative stress-driven hepatotoxicity via inhibiting ASK1/JNK and Txnip/NLRP3 inflammasome activation. Phytomedicine 71 32454347 10.1016/j.phymed.2020.153241

[91] Motawi TK, El-Maraghy SA, Kamel AS, Said SE, Kortam MA (2023) Modulation of p38 MAPK and Nrf2/HO-1/NLRP3 inflammasome signaling and pyroptosis outline the anti-neuroinflammatory and remyelinating characters of clemastine in EAE rat model. Biochem Pharmacol 209 36720356 10.1016/j.bcp.2023.115435

[92] Prince TL, Kijima T, Tatokoro M, Lee S, Tsutsumi S, Yim K et al (2015) Client proteins and small molecule inhibitors display distinct binding preferences for constitutive and stress-induced HSP90 isoforms and their conformationally restricted mutants. PLoS ONE 10(10) 26517842 10.1371/journal.pone.0141786 PMC4627809

[93] Deng NH, Zhou ZX, Liu HT, Tian Z, Wu ZF, Liu XY et al (2022) TRIMs: generalists regulating the NLRP3 inflammasome signaling pathway. DNA Cell Biol 41(3):262–275 35180350 10.1089/dna.2021.0943 PMC8972007

[94] Song Y, Qu Y, Mao C, Zhang R, Jiang D, Sun X. Post-translational modifications of Keap1: the state of the art. Vol. 11, Frontiers in cell and developmental biology. Frontiers Media SA; 2023. 10.3389/fcell.2023.1332049 PMC10801156 38259518

[95] Wan H, Cai Y, Xiao L, Ling Y, Ge L, Mo S et al (2023) JFD, a novel natural inhibitor of Keap1 alkylation, suppresses intracellular mycobacterium tuberculosis growth through Keap1/Nrf2/SOD2-mediated ROS accumulation. Oxid Med Cell Longev 10(2023):1–21 10.1155/2023/6726654 PMC9937762 36819778

[96] Fourquet S, Guerois R, Biard D, Toledano MB (2010) Activation of NRF2 by nitrosative agents and H2O2 involves KEAP1 disulfide formation. J Biol Chem 285(11):8463–8471 20061377 10.1074/jbc.M109.051714 PMC2832995

[97] Buckley BJ, Li S, Whorton AR (2008) Keap1 modification and nuclear accumulation in response to S-nitrosocysteine. Free Radic Biol Med 44(4):692–698 18062931 10.1016/j.freeradbiomed.2007.10.055 PMC2267934

[98] Um HC, Jang JH, Kim DH, Lee C, Surh YJ (2011) Nitric oxide activates Nrf2 through S-nitrosylation of Keap1 in PC12 cells. Nitric Oxide 25(2):161–168 21703357 10.1016/j.niox.2011.06.001

[99] Zhang XW, Feng N, Liu YC, Guo Q, Wang JK, Bai YZ, et al. Neuroinflammation inhibition by small-molecule targeting USP7 noncatalytic domain for neurodegenerative disease therapy. Sci Adv. 2022 Aug 12;8 (32). 10.1126/sciadv.abo0789 PMC9365288 35947662

[100] Wang L, Qu G, Gao Y, Su L, Ye Q, Jiang F et al (2018) A small molecule targeting glutathione activates Nrf2 and inhibits cancer cell growth through promoting Keap-1 *S*-glutathionylation and inducing apoptosis. RSC Adv 8(2):792–804 35538996 10.1039/c7ra11935f PMC9076930

[101] Curtis JM, Hahn WS, Long EK, Burrill JS, Arriaga EA, Bernlohr DA (2012) Protein carbonylation and metabolic control systems. Trends Endocrinol Metab 23(8):399–406 22742812 10.1016/j.tem.2012.05.008 PMC3408802

[102] Meng W, Pei Z, Feng Y, Zhao J, Chen Y, Shi W et al (2017) Neglected role of hydrogen sulfide in sulfur mustard poisoning: Keap1 S-sulfhydration and subsequent Nrf2 pathway activation. Sci Rep 7(1):9433 28842592 10.1038/s41598-017-09648-6 PMC5572733

[103] Wei S, Pei Y, Wang Y, Guan H, Huang Y, Xing T et al (2019) Role of human Keap1 S53 and S293 residues in modulating the binding of Keap1 to Nrf2. Biochimie 158:73–81 30576774 10.1016/j.biochi.2018.12.008

[104] Chen P, Smith TJ, Wu J, Siesser PF, Bisnett BJ, Khan F et al (2017) Glycosylation of Keap1 links nutrient sensing to redox stress signaling. EMBO J 36(15):2233–2250 28663241 10.15252/embj.201696113 PMC5538768

[105] Kopacz A, Kloska D, Forman HJ, Jozkowicz A, Grochot-Przeczek A, Elsevier Inc. (2020) Beyond repression of Nrf2: an update on Keap1. Free Radic Biol Med 157:63–74 32234331 10.1016/j.freeradbiomed.2020.03.023 PMC7732858

[106] Zambrano K, Barba D, Castillo K, Noboa L, Argueta-Zamora D, Robayo P et al (2022) Fighting Parkinson’s disease: the return of the mitochondria. Mitochondrion 64:34–44 35218960 10.1016/j.mito.2022.02.003

[107] Dias V, Junn E, Mouradian MM (2013) The role of oxidative stress in Parkinson’s disease. J Parkinsons Dis 3(4):461–491 24252804 10.3233/JPD-130230 PMC4135313

[108] Wei Z, Li X, Li X, Liu Q, Cheng Y (2018) Oxidative stress in Parkinson’s disease: a systematic review and meta-analysis. Front Mol Neurosci 5 10.3389/fnmol.2018.00236 PMC6041404 30026688

[109] Wang CY, Xu Y, Wang X, Guo C, Wang T, Wang ZY (2019) Dl-3-n-butylphthalide inhibits NLRP3 inflammasome and mitigates Alzheimer’s-like pathology *via* Nrf2-TXNIP-TrX axis. Antioxid Redox Signal 30(11):1411–1431 29634349 10.1089/ars.2017.7440

[110] Virhammar J, Nyholm D (2017) Levodopa-carbidopa enteral suspension in advanced Parkinson’s disease: clinical evidence and experience. Ther Adv Neurol Disord 10(3):171–187 28344656 10.1177/1756285616681280 PMC5349373

[111] Huang B, Liu J, Meng T, Li Y, He D, Ran X et al (2018) Polydatin prevents lipopolysaccharide (LPS)-induced Parkinson’s disease via regulation of the AKT/GSK3β-Nrf2/NF-κB signaling axis. Front Immunol 5:9 10.3389/fimmu.2018.02527 PMC6230593 30455692

[112] Egbujor MC, Saha S, Buttari B, Profumo E, Saso L (2021) Activation of Nrf2 signaling pathway by natural and synthetic chalcones: a therapeutic road map for oxidative stress. Expert Rev Clin Pharmacol 14(4):465–480 33691555 10.1080/17512433.2021.1901578

[113] Mandel SA (2012) Molecular mechanisms of the neuroprotective neurorescue action of multi-target green tea polyphenols. Front Biosci S4(2):286 10.2741/S286 22202078

[114] Lee JA, Son HJ, Park KD, Han SH, Shin N, Kim JH et al (2015) A novel compound ITC-3 activates the Nrf2 signaling and provides neuroprotection in Parkinson’s disease models. Neurotox Res 28(4):332–345 26233727 10.1007/s12640-015-9550-z

[115] Darabi S, Noori-Zadeh A, Abbaszadeh HA, Rajaei F, Bakhtiyari S (2019) Trehalose neuroprotective effects on the substantia nigra dopaminergic cells by activating autophagy and non-canonical Nrf2 pathways. Iranian Journal of Pharmaceutical Research 18(3):1419–1428 32641951 10.22037/ijpr.2019.2387 PMC6934986

[116] Ahuja M, Ammal Kaidery N, Yang L, Calingasan N, Smirnova N, Gaisin A et al (2016) Distinct Nrf2 signaling mechanisms of fumaric acid esters and their role in neuroprotection against 1-methyl-4-phenyl-1,2,3,6-tetrahydropyridine-induced experimental Parkinson’s-like disease. J Neurosci 36(23):6332–6351 27277809 10.1523/JNEUROSCI.0426-16.2016 PMC4899530

[117] Lee. Quercetin exerts a neuroprotective effect through inhibition of the iNOS/NO system and pro-inflammation gene expression in PC12 cells and in zebrafish. Int J Mol Med. 2011 Feb 1;27 (2). 10.3892/ijmm.2010.571 21132259

[118] Kaidery NA, Banerjee R, Yang L, Smirnova NA, Hushpulian DM, Liby KT et al (2013) Targeting Nrf2-mediated gene transcription by extremely potent synthetic triterpenoids attenuate dopaminergic neurotoxicity in the MPTP mouse model of Parkinson’s disease. Antioxid Redox Signal 18(2):139–157 22746536 10.1089/ars.2011.4491 PMC3514006

[119] Xu W, Zheng D, Liu Y, Li J, Yang L, Shang X (2017) Glaucocalyxin B alleviates lipopolysaccharide-induced Parkinson’s disease by inhibiting TLR/NF-κB and activating Nrf2/HO-1 pathway. Cell Physiol Biochem 44(6):2091–2104 29241205 10.1159/000485947

[120] Kim S, Indu Viswanath AN, Park JH, Lee HE, Park AY, Choi JW et al (2020May) Nrf2 activator via interference of Nrf2-Keap1 interaction has antioxidant and anti-inflammatory properties in Parkinson’s disease animal model. Neuropharmacology 1(167):107989 10.1016/j.neuropharm.2020.107989 32032607

[121] Nakano-Kobayashi A, Fukumoto A, Morizane A, Nguyen DT, Le TM, Hashida K, et al. Therapeutics potentiating microglial p21-Nrf2 axis can rescue neurodegeneration caused by neuroinflammation. Sci Adv. 2020 Nov 13;6 (46). 10.1126/sciadv.abc1428 PMC7673758 33188020

[122] Zhou Y, Wang X, Ying W, Wu D, Zhong P (2019) Cryptotanshinone attenuates inflammatory response of microglial cells via the Nrf2/HO-1 pathway. Front Neurosci 21 10.3389/fnins.2019.00852 PMC6712928 31496930

[123] Baird L, Dinkova-Kostova AT (2011) The cytoprotective role of the Keap1-Nrf2 pathway. Arch Toxicol 85:241–272 21365312 10.1007/s00204-011-0674-5

[124] Wardyn JD, Ponsford AH, Sanderson CM (2015) Dissecting molecular cross-talk between Nrf2 and NF-κB response pathways. Biochem Soc Trans 43(4):621–626 26551702 10.1042/BST20150014 PMC4613495

[125] Ding Y, Chen M, Wang M, Wang M, Zhang T, Park J et al (2014) Neuroprotection by acetyl-11-keto-β-boswellic acid, in ischemic brain injury involves the Nrf2/HO-1 defense pathway. Sci Rep 4(1):7002 25384416 10.1038/srep07002 PMC4227012

[126] Xu MX, Zhu YF, Chang HF, Liang Y (2016) Nanoceria restrains PM2.5-induced metabolic disorder and hypothalamus inflammation by inhibition of astrocytes activation related NF-κB pathway in Nrf2 deficient mice. Free Radic Biol Med 99:259–272 27554971 10.1016/j.freeradbiomed.2016.08.021

[127] Sivandzade F, Prasad S, Bhalerao A, Cucullo L (2019) NRF2 and NF-қB interplay in cerebrovascular and neurodegenerative disorders: molecular mechanisms and possible therapeutic approaches. Redox Biol 1:21 10.1016/j.redox.2018.11.017 PMC6302038 30576920

[128] Liu GH, Qu J, Shen X. NF-κB/p65 antagonizes Nrf2-ARE pathway by depriving CBP from Nrf2 and facilitating recruitment of HDAC3 to MafK. Biochimica et Biophysica Acta (BBA) - Molecular Cell Research. 2008 May 1;1783 (5):713–27. 10.1016/j.bbamcr.2008.01.002 18241676

[129] Garstkiewicz M, Strittmatter GE, Grossi S, Sand J, Fenini G, Werner S et al (2017) Opposing effects of Nrf2 and Nrf2-activating compounds on the NLRP3 inflammasome independent of Nrf2-mediated gene expression. Eur J Immunol 47(5):806–817 28247911 10.1002/eji.201646665

[130] Pant T, Uche N, Juric M, Zielonka J, Bai X (2024) Regulation of immunomodulatory networks by Nrf2-activation in immune cells: redox control and therapeutic potential in inflammatory diseases. Redox Biol 70 38359749 10.1016/j.redox.2024.103077 PMC10877431

[131] van Lieshout MHP, de Vos AF, Dessing MC, de Porto APNA, de Boer OJ, de Beer R et al (2018) ASC and NLRP3 impair host defense during lethal pneumonia caused by serotype 3 *Streptococcus pneumoniae* in mice. Eur J Immunol 48(1):66–79 28971472 10.1002/eji.201646554

[132] Zhou Q, Chen B, Wang X, Wu L, Yang Y, Cheng X et al (2016) Sulforaphane protects against rotenone-induced neurotoxicity in vivo: involvement of the mTOR, Nrf2 and autophagy pathways. Sci Rep 6(1):32206 27553905 10.1038/srep32206 PMC4995453

[133] Qiao J, Ma H, Chen M, Bai J (2023) Vitamin D alleviates neuronal injury in cerebral ischemia-reperfusion via enhancing the Nrf2/HO-1 antioxidant pathway to counteract NLRP3-mediated pyroptosis. J Neuropathol Exp Neurol 82(8):722–733 37403613 10.1093/jnen/nlad047

[134] Rasheed MSU, Tripathi MK, Patel DK, Singh MP (2020) Resveratrol regulates Nrf2-mediated expression of antioxidant and xenobiotic metabolizing enzymes in pesticides-induced parkinsonism. Protein Pept Lett 27(10):1038–1045 32242774 10.2174/0929866527666200403110036

[135] Xu Q, Chen Y, Chen D, Reddy MB (2024) The protection of EGCG against 6-OHDA-induced oxidative damage by regulating PPARγ and Nrf2/HO-1 signaling. Nutr Metab Insights 25 10.1177/11786388241253436 PMC11128170 38800717

[136] Zamanian MY, Terefe EM, Taheri N, Kujawska M, Tork YJ, Abdelbasset WK et al (2023) Neuroprotective and anti-inflammatory effects of pioglitazone on Parkinson’s disease: a comprehensive narrative review of clinical and experimental findings. CNS Neurol Disord Drug Targets 22(10):1453–1461 36200161 10.2174/1871527322666221005122408

[137] Sharma V, Sharma P, Singh TG (2024) Emerging role of Nrf2 in Parkinson’s disease therapy: a critical reassessment. Metab Brain Dis 40(1):70 39699763 10.1007/s11011-024-01452-2

[138] Harrison D, Billinton A, Bock MG, Doedens JR, Gabel CA, Holloway MK et al (2023) Discovery of clinical candidate NT-0796, a brain-penetrant and highly potent NLRP3 inflammasome inhibitor for neuroinflammatory disorders. J Med Chem 66(21):14897–14911 37874905 10.1021/acs.jmedchem.3c01398

[139] Kaur B, Biby S, Namme JN, More S, Xu Y, Zhang S. Biological and therapeutic significance of targeting NLRP3 inflammasome in the brain and the current efforts to develop brain-penetrant inhibitors. In 2025. p. 103–57. 10.1016/bs.apha.2024.10.004 PMC11955958 39929578

[140] D’Urso G, Thomann A, Anzures-Cabrera J, Zinnhardt B, Ricci B, Bailey L, et al. Phase Ib study design evaluating the safety, pharmacokinetics and pharmacodynamics of selnoflast, a novel NLRP3 inflammasome inhibitor in early-stage PD (P2–11.007). Neurology. 2023 Apr 25;100 (17_supplement_2).

[141] Amo-Aparicio J, Daly J, Højen JF, Dinarello CA (2023) Pharmacologic inhibition of NLRP3 reduces the levels of α-synuclein and protects dopaminergic neurons in a model of Parkinson’s disease. J Neuroinflammation 20(1):147 37349821 10.1186/s12974-023-02830-w PMC10286423

[142] Parmar DV, Kansagra KA, Momin T, Patel HB, Jansari GA, Bhavsar J et al (2023) Safety, tolerability, pharmacokinetics, and pharmacodynamics of the oral NLRP3 inflammasome inhibitor ZYIL1: first-in-human phase 1 studies (single ascending dose and multiple ascending dose). Clin Pharmacol Drug Dev 12(2):202–211 36065092 10.1002/cpdd.1162 PMC10087697

[143] Fang J, She J, Lin F, Wu JC, Han R, Sheng R et al (2022) RRx-001 exerts neuroprotection against LPS-induced microglia activation and neuroinflammation through disturbing the TLR4 pathway. Front Pharmacol 6:13 10.3389/fphar.2022.889383 PMC9020799 35462935

